# Altermagnetic spin textures: Emergent electrodynamics, quantum geometry, and probes

**DOI:** 10.1126/sciadv.aeh1411

**Published:** 2026-09-18

**Authors:** Constantin Schrade, Mathias S. Scheurer

**Affiliations:** ^1^ Hearne Institute of Theoretical Physics, Department of Physics and Astronomy, Louisiana State University, Baton Rouge, LA 70803, USA.; ^2^ Institute for Theoretical Physics III, University of Stuttgart, 70550 Stuttgart, Germany.

## Abstract

Emergent electrodynamics arising from spatially and temporally varying magnetic textures provides a framework for spin control in quantum materials. While this principle is established for ferromagnetic and antiferromagnetic textures, its consequences for altermagnets—magnetic orders with vanishing net magnetization but finite spin splitting—remain largely unexplored. In this work, we develop an effective low-energy theory of itinerant electrons coupled to smoothly varying altermagnetic spin textures. In the adiabatic regime, we show that altermagnetic textures generate additional emergent electromagnetic fields and quantum-geometric effects that are absent in conventional magnetic systems. These effects include emergent Zeeman fields that encode the structure of the altermagnetic order parameter, enabling local spin manipulation and a way to distinguish different altermagnetic orders. Moreover, we demonstrate a quantum metric–induced, spin-dependent electron lensing effect that provides a mechanism for spin-dependent transmissions and discuss the local admixture of effective odd-parity magnetic components. Our results suggest that textured altermagnets could serve as a versatile resource for spintronics functionalities and a probe of altermagnetism.

## INTRODUCTION

Spin textures play a central role in condensed matter systems, ranging from magnetic textures in spintronics (*1*, *2*) to moiré lattices with spatially varying effective spin degrees of freedom (*3*–*7*). A particularly interesting feature of such textures is the emergence of effective electrodynamics for itinerant electrons, which has been studied for ferromagnets (*8*–*14*) and antiferromagnets (*15*–*18*). Intuitively, as an electron moves through a smoothly varying texture (with a characteristic length scale much larger than the Fermi wavelength), its spin must continuously align with the effective magnetic field, which generates additional forces and modifies its dynamics. These additional contributions can be mapped to emergent electromagnetic fields and a distortion of the underlying space in which the electrons propagate. From a lattice perspective, the same physics appears as a texture-dependent quenching of hopping amplitudes set by the relative orientation of neighboring spins. Emergent electrodynamics and quantum geometry of this kind, as well as current-induced torques (*19*–*24*), provide a powerful route to controlling spin degrees of freedom for spintronics applications (*25*–*27*).

Another exciting opportunity in spintronics that emerged more recently are altermagnets (*28*–*32*). In the simplest collinear cases, these magnetically ordered states are characterized by two magnetic sublattices which are related by symmetries that are—as opposed to antiferromagnets—not translation or inversion. A direct consequence is a finite spin splitting of the electronic bands while retaining vanishing net magnetization, which makes altermagnets attractive for spintronics applications (*27*). Although spatially (e.g., domain walls) and possibly also temporally varying textures of the altermagnetic order parameter, ***n***(***r***,t), are expected to be ubiquitous in real samples, in line with recent experiments (*33*, *34*), there are only very few theoretical works studying them (*35*–*38*); these studies focus on the dynamics and form of the magnetic order parameter fields themselves, rather than on the impact on the electronic spectrum. Thus far, electronic properties of inhomogeneous altermagnets have primarily been studied in the context of atomic-scale defects (*39*–*46*), as well as in studies on strain-induced responses (*47*, *48*).

**Fig. 1.**
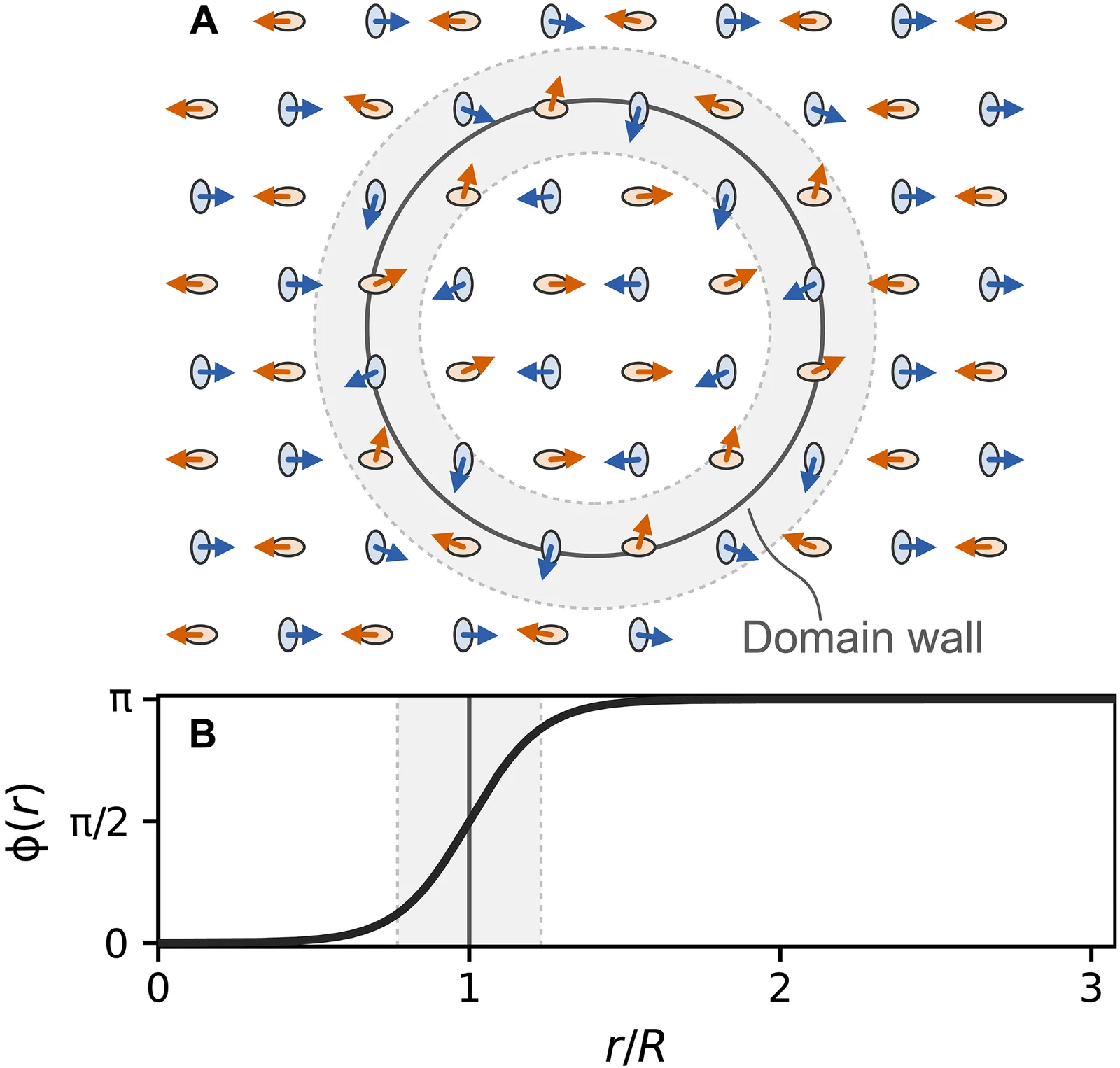
Circular Néel domain wall separating two altermagnetic domains. (**A**) Real-space schematic of the Néel vector field, n(r), on the two sublattices ($\tau_{z}=\pm$; orange and blue). Arrows indicate the local in-plane spin orientation. The shaded annulus denotes the domain wall. The solid circle marks the domain wall radius, *R*. (**B**) Radial rotation profile, ϕ(r), describing the π rotation of ***n*** across the wall. The wall center is at r=R. The illustrations are schematic and not to scale. Here, we assume a texture with a≪w≪R (*a* is the lattice constant and *w* is the wall width).

In this work, we fill this gap and develop a theory for the impact of smoothly varying altermagnetic order parameters on the electronic properties. This is particularly relevant for altermagnets where the impact of the magnetic moments on the electronic bands is nontrivial and their key defining feature. We will contrast the emergent electromagnetic fields and curved spacetime with the ferromagnetic and antiferromagnetic cases, revealing that there are novel contributions unique to altermagnets. We show that this not only allows us to identify altermagnetic order but also to distinguish between different forms (*d*- versus *g*-wave) of altermagnetic order parameters. While our theory applies to generic textures, we primarily illustrate our findings using a circular domain wall, as illustrated in Fig. 1.

## RESULTS AND DISCUSSION

### Minimal model

As a starting point, we use the following minimal model for an altermagnetic spin texture

$$\hat{H}=\varepsilon_{0,\hat{p}}\tau_{0}+t_{x,\hat{p}}\tau_{x}+t_{z,\hat{p}}\tau_{z}+J\tau_{z}n\left(r,t\right)\cdot s$$ (1)

motivated by earlier works on uniform altermagnets (*39*, *49*, *50*). Here, the Pauli matrices $\left(\tau_{x},\tau_{y},\tau_{z}\right)$ and $s={\left(s_{x},s_{y},s_{z}\right)}^{T}$ act in sublattice and spin-space, respectively. Moreover, $\varepsilon_{0,\hat{p}}$ describes the sublattice-independent contribution to the Hamiltonian with $\hat{p}=-i\hbar {\left(\partial_{x},\partial_{y}\right)}^{T}$ being the momentum operator, whereas $t_{z,\hat{p}}$ and $t_{x,\hat{p}}$ are sublattice asymmetric and intersublattice contributions. For an altermagnet, $t_{z,\hat{p}}$ transforms as a nontrivial, one-dimensional irreducible representation of the crystallographic point group. In the last term, n(r,t) describes the spatially $\left[r={\left(x,y\right)}^{T}\right]$ and temporally (*t*) varying order parameter. When n(r,t) is spatially uniform and time independent, Eq. 1 is readily diagonalized and yields the characteristic spin splitting of the bands with angular dependence determined by the functional form of $t_{z,p}$ (as will become more explicit below). In particular, the splitting will vanish for $t_{z,p}=0$, which corresponds to antiferromagnetism.

To describe the effects of the altermagnetic spin texture, it is useful to move to a frame in which the quantization axis follows the local orientation of the Néel vector. We therefore parametrize the Néel vector as ***n***(***r***,*t*) = [sinθ(***r***,*t*)cosϕ(***r***,*t*), sinθ(***r***,*t*)sinϕ(***r***,*t*), cosθ(***r***,*t*)]*^T^* and define the unitary transformation U(r,t)=$\text{exp}\left\{\frac{i}{2}\;\theta \left(r,t\right)\left[\text{sin}\phi \left(r,t\right)s_{x}-\text{cos}\phi (r,t)s_{y}\right]\right\}$. Under this transformation, the last term in Eq. 1 becomes uniform since $U^{†}\left(r,t\right)\;\left[n\left(r,t\right)\cdot s\right]U\left(r,t\right)=s_{z}$. The information on the spatial and temporal variation of the altermagnetic spin texture are now contained in spin-dependent gauge fields, A(r,t), that shift the momentum operators as $\hat{\pi }\equiv$$U^{†}\left(r,t\right)\hat{p}U\left(r,t\right)=\hat{p}-A\left(r,t\right)$ with $A\left(r,t\right)\equiv -U^{†}\left(r,t\right)\left[\hat{p}U\left(r,t\right)\right]$. The time dependence of the order parameter further generates a temporal gauge field, $A_{0}\left(r,t\right)\equiv i\hbar \;U^{†}\left(r,t\right)\left[\partial_{t}U\left(r,t\right)\right]$. The rotating frame Hamiltonian, ${\hat{H}}_{\text{rot}}=U^{†}\left(r,t\right)\hat{H}\left(t\right)U\left(r,t\right)-A_{0}\left(r,t\right)$, is then given by

$${\hat{H}}_{\text{rot}}=\varepsilon_{0,\hat{\pi }}\tau_{0}+t_{x,\hat{\pi }}\tau_{x}+t_{z,\hat{\pi }}\tau_{z}+J\tau_{z}s_{z}-A_{0}$$ (2)

Here, we emphasize that the shifted momenta still commute, $\left[{\hat{\pi }}_{i},{\hat{\pi }}_{j}\right]=U^{†}\left(r,t\right)\left[{\hat{p}}_{i},{\hat{p}}_{j}\right]U\left(r,t\right)=0$. However, as we will see in the next section, this commuting property will be violated upon projecting the rotating frame Hamiltonian onto its low-energy subspace, leading to emergent electromagnetic fields.

Last, it is instructive to introduce two examples that will guide the rest of this work. The first example is that of a *d*-wave altermagnet. Only keeping the leading power in momentum for every term, we set $\varepsilon_{0,p}=\varrho_{0}\left(p_{x}^{2}+p_{y}^{2}\right)$, $t_{x,p}=t_{x}$, and $t_{z,p}=\varrho_{z}\left(p_{x}^{2}-p_{y}^{2}\right)$. In the limit of a spatially uniform and time-independent n(r,t), this choice gives the usual dispersion of a conventional *d*-wave altermagnet. The second example is that of a *g*-wave altermagnet, where $\varepsilon_{0,p}$ and $t_{x,p}$ are chosen as before. Meanwhile, for $t_{z,p}$, we choose a *g*-wave form, $t_{z,p}={\bar{\varrho }}_{z}p_{x}p_{y}\left(p_{x}^{2}-p_{y}^{2}\right)$. At the operator level, we will use the representation ${\hat{t}}_{z,\hat{p}}={\bar{\varrho }}_{z}W\left[{\hat{p}}_{x}{\hat{p}}_{y}\left({\hat{p}}_{x}^{2}-{\hat{p}}_{y}^{2}\right)\right]$ where we have introduced the fully symmetrized “Weyl” ordering, $W\left({\hat{O}}_{1}{\hat{O}}_{2}{\hat{O}}_{3}{\hat{O}}_{4}\right)=\sum_{\pi \in S_{4}}\left[{\hat{O}}_{\pi \left(1\right)}{\hat{O}}_{\pi \left(2\right)}{\hat{O}}_{\pi \left(3\right)}{\hat{O}}_{\pi \left(4\right)}\right]/4!$ with $S_{4}$ being the symmetric group on four elements. At this point, this representation is redundant, since both the momenta and shifted momenta commute. However, it will be useful later when discussing the semiclassical limit.

### Emergent electrodynamics

Emergent electromagnetic fields are known to arise in ferromagnetic (*8*–*14*) and antiferromagnetic (*15*–*18*) spin textures. Here, we show that altermagnets support qualitatively new emergent fields. These fields provide ways to distinguish (i) altermagnets from ferro- and antiferromagnets and (ii) different altermagnetic order parameters.

As a first step to derive the emergent electromagnetic fields, it will be useful to decompose the spin-dependent gauge fields into its longitudinal and transversal components, $A_{\mu }\left(r,t\right)=A_{\mu }^{∥}\left(r,t\right)+A_{\mu }^{⊥}\left(r,t\right)$ with $A_{\mu }^{∥}\left(r,t\right)\propto s_{z}$, $A_{\mu }^{⊥}\left(r,t\right)\propto s_{x,y}$, and μ∈{0,x,y}. This decomposition allows us to introduce momentum operators shifted by the longitudinal gauge field, ${\hat{\Pi }}_{i}={\hat{p}}_{i}-A_{i}^{∥}\left(r,t\right)$ so that ${\hat{\pi }}_{i}={\hat{\Pi }}_{i}-A_{i}^{⊥}\left(r,t\right).$ Also, we introduce ${\hat{\Pi }}_{0}=i\hbar \partial_{t}-A_{0}^{∥}\left(r,t\right)$. One important feature of these newly defined momentum operators is that they do not generally commute. Instead, we find that $\left[{\hat{\Pi }}_{x},{\hat{\Pi }}_{y}\right]=i\hbar B\left(r,t\right)$ and $\left[{\hat{\Pi }}_{0},{\hat{\Pi }}_{i}\right]=i\hbar E_{i}\left(r,t\right)$ with the effective spin-dependent orbital (out-of-plane) magnetic fields and (in-plane) electric field components

$$\begin{array}{c} B\left(r,t\right)=-s_{z}\;\frac{\hbar }{2}\;n\left(r,t\right)\cdot \left[\partial_{x}n\left(r,t\right)\times \partial_{y}n\left(r,t\right)\right],\\ E_{i}\left(r,t\right)=-s_{z}\;\frac{\hbar }{2}\;n\left(r,t\right)\cdot \left[\partial_{t}n\left(r,t\right)\times \partial_{i}n\left(r,t\right)\right] \end{array}$$ (3)

We will now show that the itinerant electrons in our altermagnetic spin texture will effectively be subject to these B(r,t) and $E_{i}\left(r,t\right)$ fields as well as to an emergent Zeeman field. For this purpose, we will assume that J>0 is a sufficiently large energy scale so that the low-energy states of our altermagnetic spin texture lie in the subspace where $S\equiv \tau_{z}s_{z}$ has eigenvalue −1 and define the associated projection operator $P_{-}=\left(1-S\right)/2$. We will next study our rotating frame Hamiltonian in Eq. 2 upon being projected onto the S=−1 while, for now, neglecting virtual transitions to the high-energy S=+1 subspace.

We initially focus on our first example of the *d*-wave altermagnet. In this case, we find that the effective Hamiltonian (to _zeroth_ order in 1/J), ${\hat{H}}_{\text{rot}}^{\left(0\right)}=P_{-}{\hat{H}}_{\text{rot}}P_{-}$, evaluates to

$${\hat{H}}_{\text{rot}}^{\left(0\right)}=\varepsilon_{0,\hat{\Pi }}\sigma_{0}+t_{z,\hat{\Pi }}\sigma_{z}+\varrho_{0}V_{0}\sigma_{0}+\varrho_{z}V_{z}\sigma_{z}-\sigma_{z}a_{0}^{∥}$$ (4)

Here, we already expressed the Hamiltonian as an effective two-band model using the Pauli matrices $\sigma_{j}$ acting on the low-energy degrees of freedom with basis states (τ=+,s=↓) and (τ=−,s=↑). In this expression, $s_{z}$ in $\hat{\Pi }$ is implicitly replaced by $-\sigma_{z}$. Furthermore, $a_{0}\left(r,t\right)=\left(\hbar /2\right)\left[\hat{z}\cdot \left(n\times \partial_{t}n\right)\right]/\left(1+n_{z}\right)$ is the temporal component of the projected longitudinal gauge field. Moreover, we have defined the scalar potentials

$$V_{0}=\hbar^{2}\delta^{\mathrm{ij}}g_{\mathrm{ij}}\left(r,t\right),\;V_{z}=\hbar^{2}\eta^{\mathrm{ij}}g_{\mathrm{ij}}\left(r,t\right)$$ (5)

Here, $\eta^{\mathrm{ij}}=\text{diag}{\left(1,-1\right)}_{\mathrm{ij}}=\frac{1}{2!}\frac{1}{\varrho_{z}}{\frac{\partial^{2}t_{z,p}}{\partial p_{i}\;\partial p_{j}}|}_{p=0}$, $g_{\mathrm{ij}}\left(r,t\right)=\partial_{i}n\left(r,t\right)\cdot$ $\partial_{j}n\left(r,t\right)/4$ is the quantum metric associated with the spin texture [i.e., of the eigenstates $\psi_{r}\in {\mathbb{C}}^{2}$ of n(r,t)⋅s], and summation over the repeated indices i,j∈{x,y} is left implicit.

Two comments about this result for the *d*-wave altermagnetic texture are in order: First, the itinerant electrons experience emergent orbital magnetic and electric fields, B(r,t) and $E_{i}\left(r,t\right)$, as they move through the texture. These fields have opposite signs for the two sublattices due to the prefactor $s_{z}$ (which is equivalent to $-\tau_{z}$ in $P_{-}$) and require noncoplanar textures in space (B) or space-time ($E_{i}$). However, we note that both fields are not genuinely unique to altermagnets since they arise even in the antiferromagnet (*15*) limit, $t_{z,p}\to 0$, and hence not our main subject of interest here.

Second, the itinerant electrons also experience an emergent Zeeman field, $V_{z}$. This Zeeman field is unique to the altermagnet, since it vanishes in the limit when $\varrho_{z}\to 0$. In particular, it is of geometric origin as it involves the quantum metric. To further explain the effects of this Zeeman field, we move to the principal axis frame of the quantum metric through a rotation, $g=R_{\psi }\text{diag}\left(\lambda_{1},\lambda_{2}\right)R_{\psi }^{T}$ where $R_{\psi }$ is a 2*d* rotation by an angle, ψ, and $\lambda_{1,2}\geq 0$ are the eigenvalues of the quantum metric tensor. Then, $V_{z}=\hbar^{2}\left(\lambda_{1}-\lambda_{2}\right)\text{cos}2\psi$. Hence, $V_{z}$ measures how the principal axes of the texture are oriented relative to *d*-wave altermagnetic order parameter. Such an effect does not occur for $V_{0}=\hbar^{2}\left(\lambda_{1}+\lambda_{2}\right)$, since it does not depend on ψ.

**Fig. 2.**
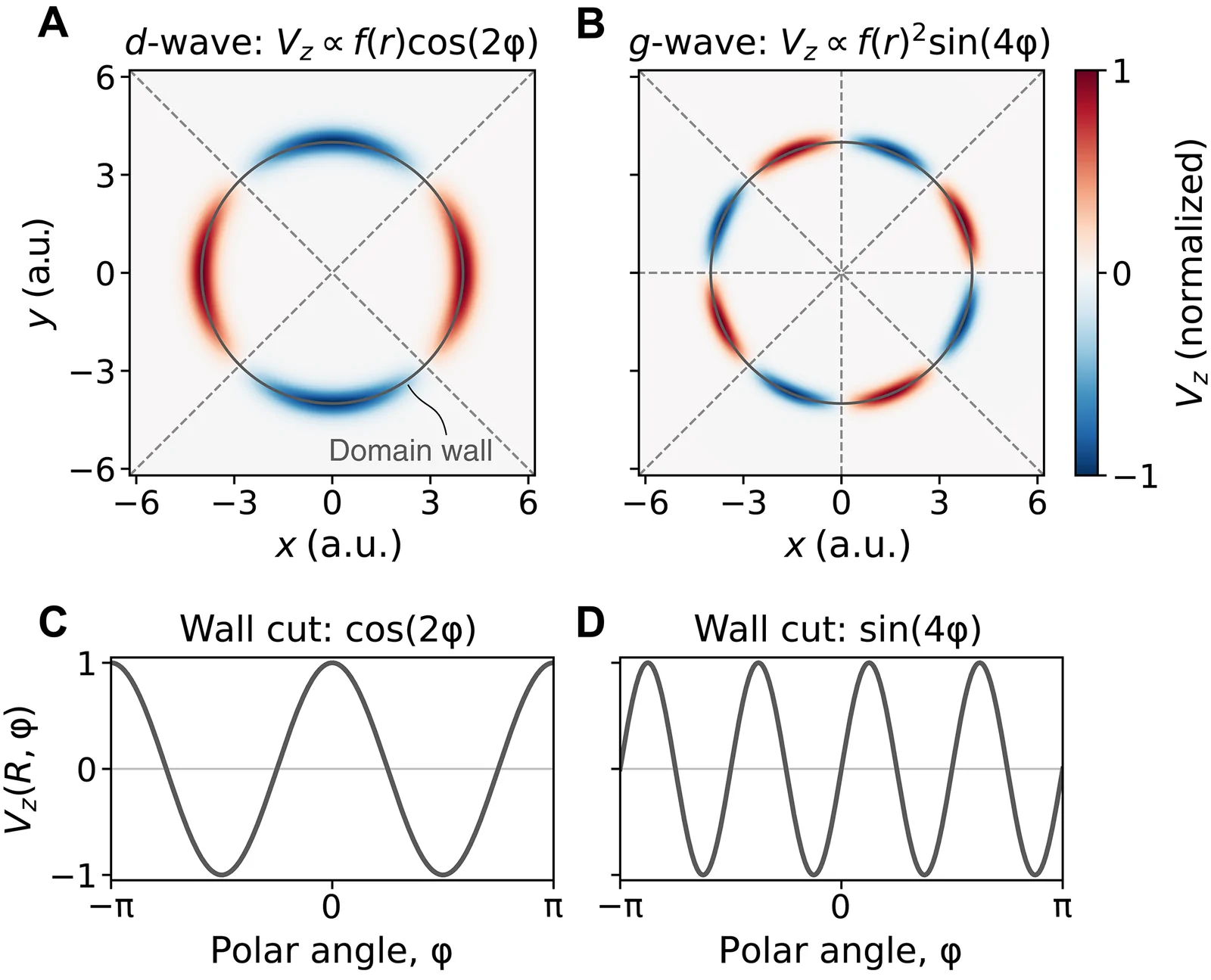
Multipolar emergent Zeeman fields from altermagnetic domain walls. (**A**) Emergent Zeeman field, $V_{z}\left(r\right)$ (normalized by its maximum), for a circular domain wall in a *d*-wave altermagnet, showing a quadrupolar pattern localized at the wall. (**B**) Corresponding result for a *g*-wave altermagnet, showing an octupolar pattern. (**C** and **D**) Angular cuts, $V_{z}\left(R,\varphi \right)$, at the wall radius, showing the cos(2φ) and sin(4φ) dependence. The multipolar form of $V_{z}$ provides a novel probe for altermagnetic order.

Another important feature of the emergent Zeeman field is that it produces a quadrupolar moment that inherits the structure of the altermagnetic order through $\eta^{\mathrm{ij}}$. To explain this feature, we consider a static radial domain wall with θ(r)=π/2 and $\phi \left(r\right)=\left(\pi /2\right)\text{tanh}\left[\left(r-R\right)/w\right]$ where *R* is the domain radius, w≪R the domain wall width, and r=∣r∣, see Fig. 1. In this case, $\lambda_{1}=\phi^{\prime }{\left(r\right)}^{2}/4$, $\lambda_{2}=0$, and ψ=φ is the polar angle. Hence, $V_{z}=f\left(r\right)\text{cos}2\varphi$ with $f\left(r\right)=\left(\hbar^{2}/4\right)\phi^{\prime }{\left(r\right)}^{2}$ realizes a “four-lobe” quadrupolar pattern near the domain wall boundary, which we illustrate in Fig. 2 (A and C). This quadrupole moment leads to spin accumulation near the domain wall.

An interesting question is whether such multipolar moments allow us to distinguish between different altermagnetic order parameters. To answer this question, we consider the previously introduced example of a *g*-wave altermagnet. In this case, the effective Hamiltonian (again to _zeroth_ order in 1/J) takes on the form

$${\hat{H}}_{\text{rot}}^{\left(0\right)}=\varepsilon_{0,\hat{\Pi }}\sigma_{0}+\left(t_{z,\hat{\Pi }}+\delta t_{z,\hat{\Pi }}\right)\sigma_{z}+\varrho_{0}V_{0}\sigma_{0}+{\bar{\varrho }}_{z}V_{z}\sigma_{z}$$ (6)

Here, $\delta t_{z,\hat{p}}=6\hbar^{2}{\bar{\varrho }}_{z}T^{\text{ijkl}}W\left[{\hat{p}}_{i}{\hat{p}}_{j}g_{\mathrm{kl}}\left(r,t\right)\right]$ is a correction term that is quadratic in the momenta, with $T^{\text{ijkl}}=\frac{1}{4!}\frac{1}{{\bar{\varrho }}_{z}}{\left.\frac{\partial^{4}t_{z,p}}{\partial p_{i}\partial p_{j}\partial p_{k}\partial p_{l}}\right|}_{p=0}$ denoting a totally symmetric rank 4 tensor and W is the previously introduced Weyl ordering. The emergent Zeeman field is given by

$$V_{z}=\hbar^{4}T^{\text{ijkl}}g_{\mathrm{ij}}\left(r,t\right)g_{\mathrm{kl}}\left(r,t\right)$$ (7)

To contrast this emergent Zeeman field with the *d*-wave example, let us again consider a radial domain wall. In the *g*-wave case, we find that $V_{z}=\left(\hbar^{4}/64\right)\phi^{\prime }{\left(r\right)}^{4}\text{sin}4\varphi$, which realizes an eight-lobe octupolar pattern localized near the domain-wall boundary, see Fig. 2 (B and D). The multipolar pattern from the emergent Zeeman field thus allows us to distinguish between different altermagnetic order parameters. In general, we expect the octupolar pattern in the *g*-wave case to be weaker since $V_{z}\propto \hbar^{4}$, not $V_{z}\propto \hbar^{2}$ as in the *d*-wave case. However, it should still be relevant at sharper domain walls where $\phi^{\prime }{\left(r\right)}^{4}$ is sizable. In section S4, we provide an estimate for the size of the emergent Zeeman field, for both a *d*-wave and *g*-wave altermagnet.

We note that the emergence of $V_{z}$ in the altermagnet and the lack thereof in the antiferromagnetic limit can be intuitively understood by symmetry. To this end, consider a specific point close to or right at the domain-wall boundary. While fourfold rotational symmetry $C_{4z}$ is broken locally, translational symmetry tangential to the domain wall is preserved. Since elementary translations guarantee that the magnetization vanishes in the collinear antiferromagnet, we obtain *V_z_* = 0 in this case. When $\varrho_{z}\neq 0$, leading to an altermagnet, the two sublattices are not related by a translational symmetry anymore, and it is $C_{4z}$ or the diagonal reflection symmetry $\sigma_{d}$ (combined with time-reversal Θ) that set the magnetization to zero in the homogeneous limit. For a generic point on the domain wall, both of these symmetries are broken such that a finite magnetization can develop, corresponding to $V_{z}\neq 0$ in our effective theory.

Within this picture, we can also immediately understand why the zeros of $V_{z}$ are located on the dashed lines in Fig. 2A: These are the points where the domain wall preserves the $\sigma_{d}$ symmetry, leading to *V_z_* = 0. Our argument further demonstrates that the emergent Zeeman field and associated net magnetization is not a specific feature of our model but generally expected to hold for any altermagnet with the same symmetries. We can also phrase the conditions in more general terms: let $S_{p}$ be the set of symmetries that guarantee that the total magnetization vanishes in the homogeneous altermagnet. We then expect a net magnetization and hence nonzero emergent Zeeman field if and only if the domain wall locally breaks all symmetries in $S_{p}$. For instance, in the case of our *g*-wave altermagnet, we have $S_{p}=\left\{\sigma_{d}\Theta ,\sigma_{v}\Theta \right\}$. On a circular domain wall, both of these symmetries are locally broken everywhere accept along the directions indicated by the dashed gray lines in Fig. 2B.

### Scanning SQUID protocol

We now propose a transport-based protocol for detecting the emergent Zeeman potential, $V_{z}\left(r\right)$, with scanning SQUID microscopy. The key idea is that $V_{z}\left(r\right)$ locally changes the electronic dispersion and therefore produces an anisotropic correction to the charge conductivity. When a current is driven through the sample, this anisotropy changes the current density in a spatially dependent way. The resulting nonuniform current produces an out-of-plane Oersted field, which can be measured as a spatially varying SQUID flux signal. This approach builds on scanning SQUID experiments that have imaged current-induced flux signals in other quantum materials (*51*, *52*).

**Fig. 3.**
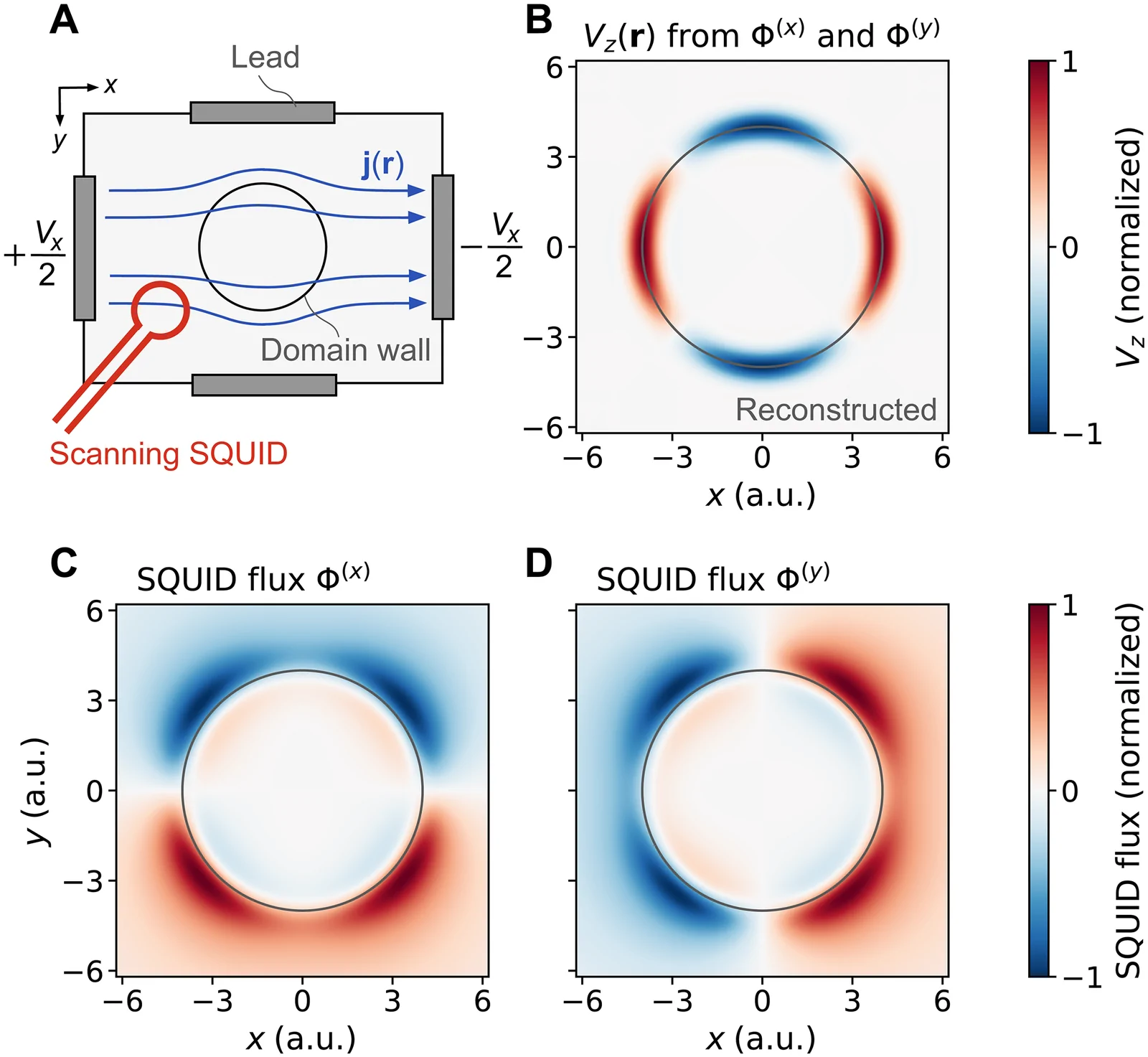
Scanning SQUID detection of the emergent Zeeman field. (**A**) Proposed measurement geometry. A four-terminal device is voltage biased along either *x* or *y*, while a scanning SQUID measures the flux produced by the induced current distribution above a circular *d*-wave domain wall. (**B**) Reconstructed emergent Zeeman field, $V_{z}\left(r\right)$, obtained from the two flux maps $\Phi^{\left(x\right)}$ and $\Phi^{\left(y\right)}$. (**C** and **D**) Corresponding SQUID flux signals for current applied along *x* and *y*. Their different spatial structure arises from the anisotropic conductivity induced by $V_{z}\left(r\right)$.

Our proposed setup is shown in Fig. 3A. It consists of a four-terminal device in which an external electric field can be applied along two orthogonal directions, *x* and *y*. A scanning SQUID then detects the magnetic flux that results from the induced current distribution. For concreteness, we focus on a circular planar domain wall in a *d*-wave altermagnet, with the principal axes of the *d*-wave order parameter aligned with the *x* and *y* directions.

We begin by applying an electric field along either the *x* or *y* direction, denoted by $E_{x}$ or $E_{y}$. The resulting current density is $j^{\left(a\right)}\left(r\right)=\sigma \left(r\right)E^{\left(a\right)}\left(r\right)$ with $E^{\left(a\right)}\left(r\right)=E_{a}\hat{a}-\nabla \phi_{a}\left(r\right)$ and a=x,y denotes the chosen electric field direction. Here, $\phi_{a}\left(r\right)$ is the electrochemical potential generated within the sample to enforce current conservation, $\nabla \cdot j^{\left(a\right)}\left(r\right)=0$. Within the Drude relaxation time approximation, the conductivity tensor in the zero-temperature limit takes the form (see section S5)

$$\sigma_{\mathrm{ij}}\left(r\right)=\chi_{0}\delta_{\mathrm{ij}}-\chi_{0}^{\prime }V_{0}\left(r\right)\delta_{\mathrm{ij}}-\chi_{z}V_{z}\left(r\right)\eta_{\mathrm{ij}}$$ (8)

This is the first result for our measurement algorithm. Here, the first term is the uniform isotropic conductivity, while $V_{0}\left(r\right)$ and $V_{z}\left(r\right)$ in the second and third terms produce local corrections. The contribution from $V_{z}\left(r\right)$ enters with opposite sign for a=x,y, which is what allows it to be isolated experimentally. The coefficients in Eq. 8 are $\chi_{0}=\left(e^{2}\tau_{\text{rel}}/\pi \hbar^{2}\right)\;\varrho_{0}\mu /{\left(\varrho_{0}^{2}-\varrho_{z}^{2}\right)}^{1/2}$, $\chi_{0}^{\prime }=$$\left(e^{2}\tau_{\text{rel}}/\pi \hbar^{2}\right)\;\varrho_{0}^{2}/{\left(\varrho_{0}^{2}-\varrho_{z}^{2}\right)}^{1/2}$, and $\chi_{z}=\left(e^{2}\tau_{\text{rel}}/\pi \hbar^{2}\right)\;\varrho_{z}^{2}/$${\left(\varrho_{0}^{2}-\varrho_{z}^{2}\right)}^{1/2}$, where $\tau_{\text{rel}}$ is the relaxation time and μ is the chemical potential.

The nonuniform current density generates an Oersted field that can be detected by the scanning SQUID. Since the pickup loop is sensitive to the out-of-plane field, we focus on the *z* component. It is convenient to work in momentum space, with r=(x,y) and $q=\left(q_{x},q_{y}\right)$. From the Biot-Savart law, the Oersted field at height *h* above the sample is $B_{z}\left(q,h\right)=\frac{\mu_{0}}{2}\;e^{-\mathrm{qh}}\;i\;\hat{z}\cdot \left[q\times j\left(q\right)\right]/q$ where $\mu_{0}$ is the vacuum permeability. For the two chosen electric field directions, *x* or *y*, this expression gives

$$\begin{array}{c} B_{z}^{\left(x\right)}\left(q,h\right)=\frac{\mu_{0}}{2}e^{-\mathrm{qh}}\frac{{\mathrm{iq}}_{y}}{q}E_{x}\left[\chi_{0^{\prime }}V_{0}\left(q\right)+\chi_{z}V_{z}\left(q\right)\right],\\ B_{z}^{\left(y\right)}\left(q,h\right)=\frac{\mu_{0}}{2}e^{-\mathrm{qh}}\frac{{\mathrm{iq}}_{x}}{q}E_{y}\left[-\chi_{0^{\prime }}V_{0}\left(q\right)+\chi_{z}V_{z}\left(q\right)\right] \end{array}$$ (9)

This is the second result for our measurement algorithm. It shows that the Oersted field is determined directly by $V_{0}\left(r\right)$ and $V_{z}\left(r\right)$. In particular, the $V_{0}$ contribution appears with opposite sign for the two electric field orientations, so it can be eliminated by combining the two measurements.

The SQUID does not measure $B_{z}$ directly but instead the flux through its pickup loop. For a SQUID centered at position R, the measured flux is $\Phi^{\left(a\right)}\left(R\right)=\int d^{2}r\;P\left(R-r\right)B_{z}^{\left(a\right)}\left(r,h\right)$ or, in momentum space, $\Phi^{\left(a\right)}\left(q\right)=P\left(q\right)B_{z}^{\left(a\right)}\left(q,h\right)$. Here, R is the coordinate of the SQUID in the *xy* plane and *P* is the point-spread function of the pickup loop.

The measurement algorithm then proceeds in three steps: First, one measures the flux maps $\Phi^{\left(x\right)}\left(R\right)$ and $\Phi^{\left(y\right)}\left(R\right)$ and Fourier transforms them to obtain $\Phi^{\left(x\right)}\left(q\right)$ and $\Phi^{\left(y\right)}\left(q\right)$. Second, combining the two datasets removes the $V_{0}$ contribution and gives

$$V_{z}\left(q\right)=\frac{e^{\mathrm{qh}}}{\mu_{0}\chi_{z}P\left(q\right)}\left[\frac{q}{{\mathrm{iq}}_{y}}\frac{\Phi^{\left(x\right)}\left(q,h\right)}{E_{x}}+\frac{q}{{\mathrm{iq}}_{x}}\frac{\Phi^{\left(y\right)}\left(q,h\right)}{E_{y}}\right]$$ (10)

Last, an inverse Fourier transform gives the emergent Zeeman field in position space, $V_{z}\left(r\right)=\int \frac{d^{2}q}{{\left(2\pi \right)}^{2}}e^{iq\cdot r}V_{z}\left(q\right)$. The procedure is illustrated in Fig. 3 (B to D) for a circular domain wall. We estimate the required flux sensitivity to be compatible with current sensing capabilities of scanning SQUIDs, see section S5.

### Geometry and electron dynamics

So far, we have focused on the leading corrections in 1/J to describe the altermagnetic texture. At this zeroth-order 1/J, the quantum metric, $g_{\mathrm{ij}}\left(r,t\right)$, enters only through the scalar potentials, $V_{0}$ and $V_{z}$. A natural question is if the metric can also modify the electron dynamics through the kinetic energy.

To address this question, we consider a time-independent *d*-wave altermagnetic texture. Let us begin by assuming that the intersublattice hybridization is the smallest energy scale and set *t_x_* = 0, which will allow for a systematic presentation of the findings and discussion of the different contributions. The first-order correction to the effective Hamiltonian in the rotating frame is then ${\hat{H}}_{\text{rot}}^{\left(1\right)}=-\Lambda^{†}\Lambda /2J$, where $\Lambda =P_{-}{\hat{H}}_{\text{rot}}P_{+}$ describes virtual transitions between the low-energy S=−1 and the high-energy S=+1 subspace. The full effective Hamiltonian up to first order in 1/J is given by ${\hat{H}}_{\text{rot}}^{\left(\text{eff}\right)}={\hat{H}}_{\text{rot}}^{\left(0\right)}+{\hat{H}}_{\text{rot}}^{\left(1\right)}$.

We now evaluate ${\hat{H}}_{\text{rot}}^{\left(1\right)}$ semiclassically; the full quantum mechanical operator expression is given in the Supplementary Materials. Here, we use a Wigner-Weyl formulation (*53*), which maps operators to phase-space functions and replaces operator products by Moyal products. Keeping terms up to order $\hbar^{0}$ in the expansion of Moyal products corresponds to the semiclassical approximation. In this case, the correction to the Hamiltonian function is

$$H_{\text{rot}}^{\left(1\right)}=-\frac{2\hbar^{2}}{J}\;p_{i}\left[K^{\mathrm{ij}}\;g_{j\ell }\left(r\right)\;K^{\ell m}\right]p_{m}$$ (11)

Here, $K^{\mathrm{ij}}=\varrho_{0}\delta^{\mathrm{ij}}+\varrho_{z}\sigma_{z}\eta^{\mathrm{ij}}$ is the kinetic energy tensor of the *d*-wave altermagnet.

**Fig. 4.**
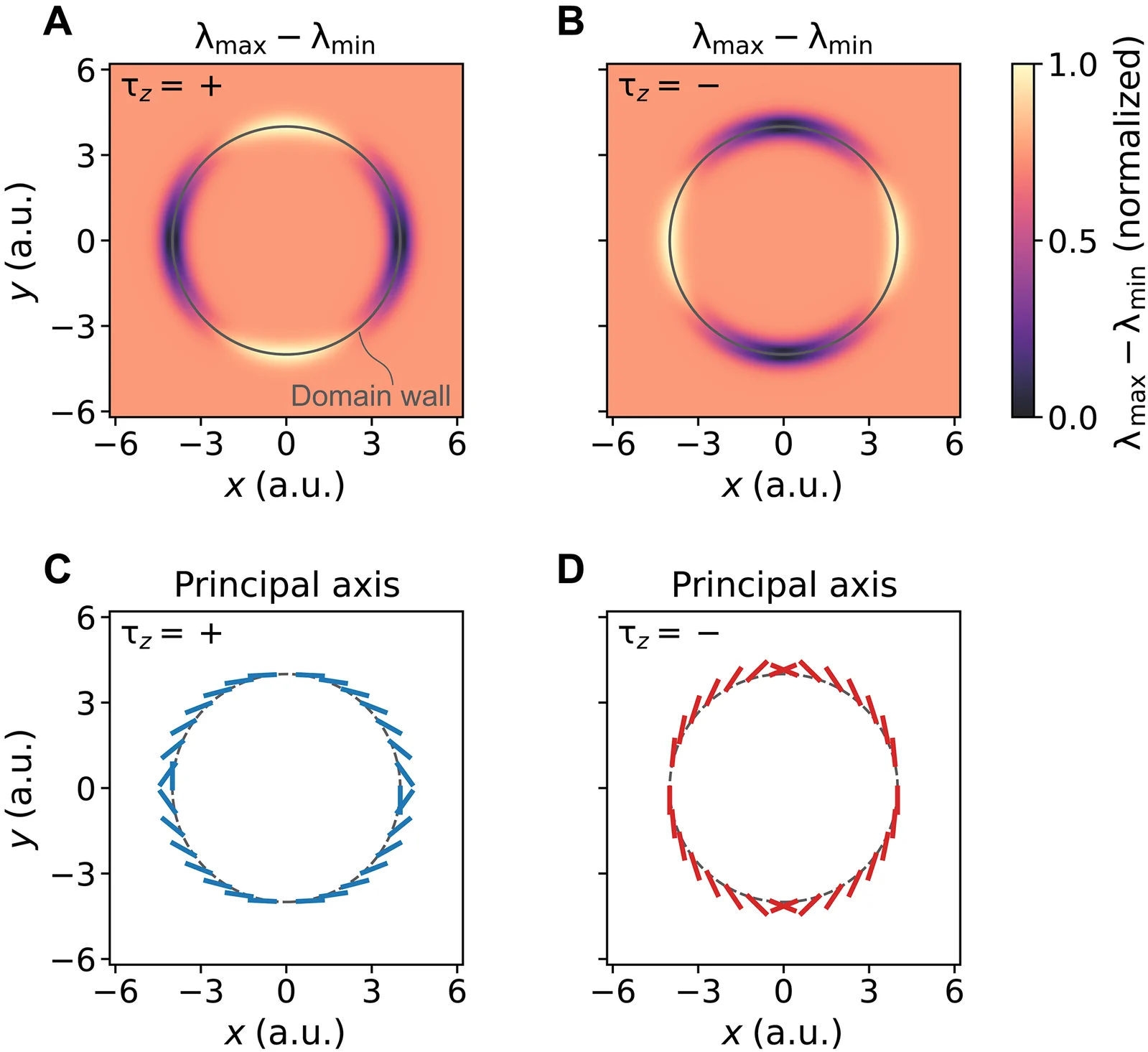
Texture-induced effective metric near a circular domain wall. (**A** and **B**) Spatial map of $\lambda_{\text{max}}-\lambda_{\text{min}}$ for $\tau_{z}=\pm$, where $\lambda_{\text{max}/\text{min}}\left(r\right)$ are the eigenvalues of the metric tensor, $g_{\text{eff},\tau }\left(r\right)$, that enters the kinetic energy. At fixed r, the local dispersion forms an ellipse in momentum space and $\lambda_{\text{max}}-\lambda_{\text{min}}$ quantifies its elongation. (**C** and **D**) Major axis of the ellipse (eigenvector of $\lambda_{\text{max}}$) evaluated along the wall, which sets the orientation of the local dispersion.

This result has a geometric interpretation. To explain this geometric interpretation, we focus on coplanar textures, so that the longitudinal gauge potential $A_{i}^{∥}\left(r\right)$ gives rise to a pure gauge and can be omitted. For a fixed sublattice τ=σ=±, the semiclassical spectrum is then given by

$$\varepsilon_{\tau }\left(p,r\right)=p_{i}\left[g_{\text{eff},\tau }^{\mathrm{ij}}\left(r\right)\right]p_{j}+V_{\tau }\left(r\right)$$ (12)

with an effective metric $g_{\text{eff},\tau }^{\mathrm{ij}}\left(r\right)=K_{\tau }^{\mathrm{ij}}-\frac{2\hbar^{2}}{J}{\left[K_{\tau }g\left(r\right)K_{\tau }\right]}^{\mathrm{ij}}$, kinetic tensor $K_{\tau }^{\mathrm{ij}}=\varrho_{0}\delta^{\mathrm{ij}}+\tau \varrho_{z}\eta^{\mathrm{ij}}$, and total scalar potential $V_{\tau }=\varrho_{0}V_{0}+\tau \varrho_{z}V_{z}$. Hence, the quantum metric gives rise to a sublattice-dependent “deformation” of the kinetic energy, see Fig. 4 for an illustration of the properties of the effective metric around a circular domain wall. Equivalently, electrons propagate as if moving in a curved space whose geometry is determined jointly by texture gradients (through $g_{\mathrm{ij}}$) and by the altermagnetic order (through $\eta^{\mathrm{ij}}$).

The same geometric picture follows from the equations of motion. Treat $r_{i}$ and $p_{j}$ as phase-space variables with Poisson brackets $\left\{r_{i},p_{j}\right\}=\delta_{\mathrm{ij}}$. Hamilton’s equations are ${\dot{r}}_{i}=\partial \varepsilon_{\tau }/\partial p_{i}$ and ${\dot{p}}_{i,\tau }=-\partial \varepsilon_{\tau }/\partial r_{i}$. Eliminating the canonical momenta leads to

$${\ddot{r}}^{i}+\Gamma_{\mathrm{jk},\tau }^{i}\left(r\right){\dot{r}}^{j}{\dot{r}}^{k}=-2g_{\text{eff},\tau }^{\mathrm{ij}}\partial_{j}V_{\tau }$$ (13)

Here, the Christoffel symbols are $\Gamma_{\mathrm{jk},\tau }^{i}\left(r\right)=\frac{1}{2}{\left(g_{\text{eff},\tau }\right)}^{i\ell }\left[\partial_{j}g_{\tau ,k\ell }^{\text{eff}}+\right.$$\left.\partial_{k}g_{\tau ,j\ell }^{\text{eff}}-\partial_{\ell }g_{\tau ,\mathrm{jk}}^{\text{eff}}\right]$ with $g_{\tau ,\mathrm{ij}}^{\text{eff}}={\left(g_{\text{eff},\tau }^{-1}\right)}_{\mathrm{ij}}$. Equation 13 shows that a semiclassical electron wave packet is subject to the following effects: First, a force from the total scalar potential, $V_{\tau }$. Second, a purely geometric contribution encoded in the Christoffel symbols of the effective metric. Third, for noncoplanar textures, an additional Lorentz-like force, $\propto \epsilon_{\mathrm{jk}}B_{\tau }{\dot{r}}^{k}$ with opposite signs for the two τ sectors, where $B_{\tau }=\tau \hbar \;n\cdot \left[\partial_{x}n\times \partial_{y}n\right]/2$. This term vanishes for the coplanar textures considered here but becomes finite when the spin texture acquires a scalar spin chirality. All of these contributions differ substantially from their ferromagnetic analog (*14*) and also lead to crucial differences between altermagnets and antiferromagnets, which we will next discuss in the context of a circular domain wall inhomogeneity.

### Altermagnetic lensing

The transport of electrons through the altermagnetic texture will provide a bending of electron trajectories, similar to the “lensing effect” in optics. We will focus on static, coplanar textures ($B_{\tau }=0$ and $E_{i}=0$), and discuss two cases: (i) lensing due to the scalar potential, $V_{\tau }$, (ii) lensing due to effective metric, $g_{\tau ,\mathrm{ij}}^{\text{eff}}$. Here, (i) corresponds to the situation when *J* is sufficiently large and electron speed is “slow,” while (ii) corresponds to fast electrons and moderately large *J* so that metric corrections to the kinetic energy become relevant. To see this, suppose we effectively increase the speed of electrons by rescaling the time coordinate, t→t/α for some α≥1. Then, $\dot{r}\to \alpha \dot{r}$ and $\ddot{r}\to \alpha^{2}\ddot{r}$. Hence, Eq. 13 reads ${\ddot{r}}^{i}+\Gamma_{\mathrm{jk},\tau }^{i}{\dot{r}}^{j}{\dot{r}}^{k}=-\left[2g_{\text{eff},\tau }^{\mathrm{ij}}\partial_{j}V_{\tau }\right]/\alpha^{2}$, and the effects of the scalar potential get suppressed upon increasing α or, equivalently, the speed of the electrons (*14*).

First, let us consider (i), lensing due to the scalar potential, $V_{\tau }$, and focus on the previously introduced radial domain wall example. In this case, $V_{z}\left(r\right)=f\left(r\right)\text{cos}2\varphi$ gives rise to a force with a tangential component $-\frac{1}{r}\partial_{\varphi }V_{z}=\frac{2f\left(r\right)}{r}\text{sin}2\varphi$ and opposite sign for the two sublattices. This force field is localized near the domain wall where f(r)≠0. As a result, an electron ray would propagate along a straight line away from the domain wall. At the wall, the ray receives a “kick” from the force field that leads to a change in its slope. This change in slope will depend on the sublattice/spin and the orientation of the ray relative to the crystal axis of the altermagnet; in particular, the kick vanishes along the crystal axis when φ=0,π/2 and hence sin2φ=0.

To further illustrate this result, we can compute the momentum transfer by solving the equations of motion. Consider a ray injected with a group velocity ${v_{\text{in},\tau }≃\nabla_{p}\varepsilon_{\tau }^{\left(0\right)}\left(p\right)|}_{p=p_{\text{in}}}$ with $\varepsilon_{\tau }^{\left(0\right)}\left(p\right)=\varepsilon_{0,p}+\tau t_{z,p}$ that hits the domain wall at a point $r_{0}=R\hat{r}\left(\varphi_{0}\right)$ where $\hat{r}=\left(\text{cos}\varphi ,\text{sin}\varphi \right)$ is the radial unit vector and $\varphi_{0}$ is the impact angle. The radial velocity component at the point of impact is $v_{r,\tau }=v_{\text{in},\tau }\cdot \hat{r}\left(\varphi_{0}\right)$. To obtain a compact analytical expression, let us first study our equations of motion in the limit of a thin domain wall, where *w* is the shortest length scale. We find that the tangential momentum is deflected by an amount

$$\Delta p_{\varphi ,\tau }≃\tau \frac{\hbar^{2}\varrho_{z}\pi^{2}}{6\mathrm{wR}}\frac{\text{sin}2\varphi_{0}}{v_{r,\tau }}$$ (14)

**Fig. 5.**
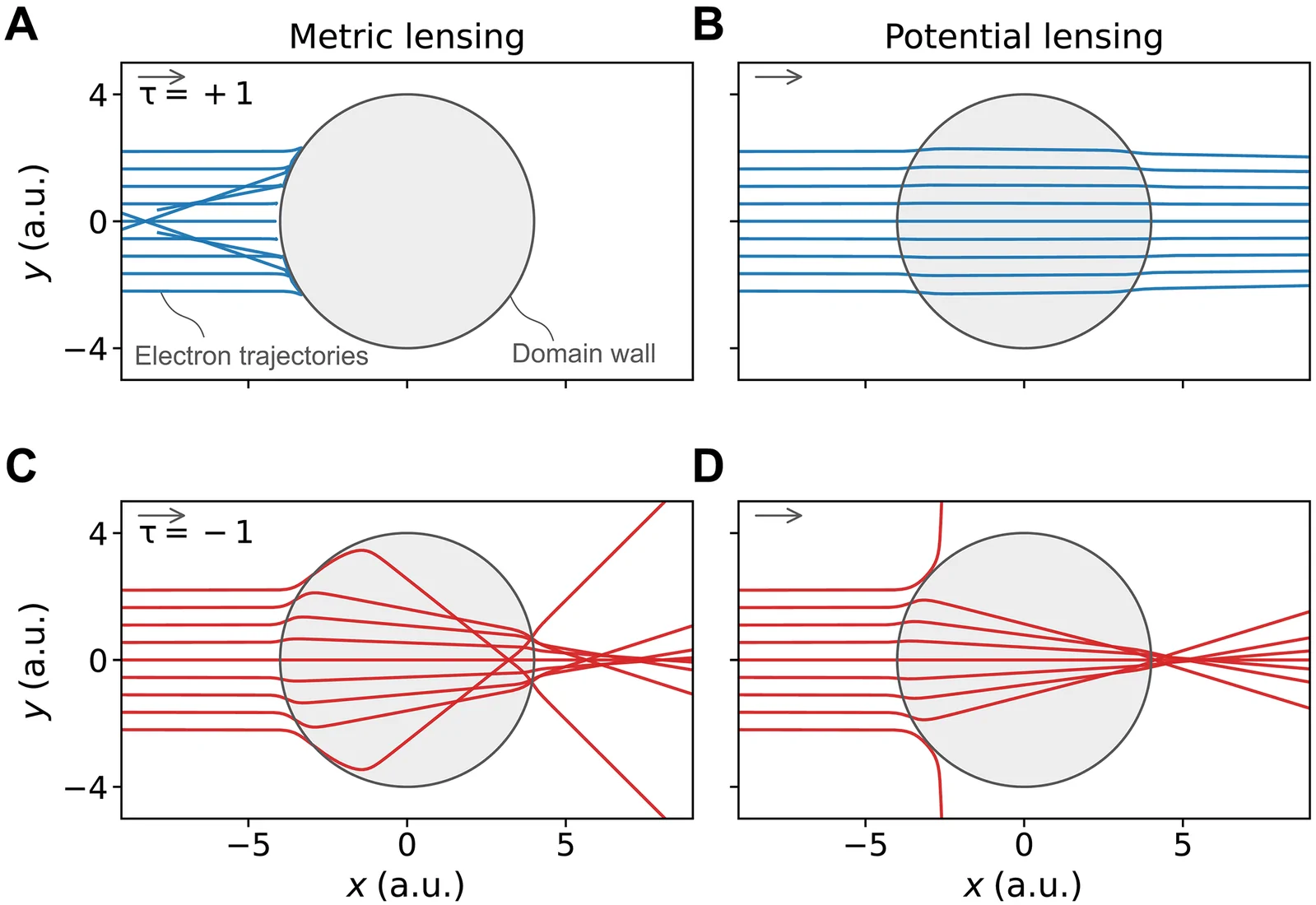
Altermagnetic domain wall as a sublattice-selective electron lens. Semiclassical trajectory incident from the left on a circular domain wall (gray disk), with τ=+1 [blue, (A) and (B)] and τ=−1 [red, (C) and (D)]. (**A** and **C**) metric lensing from the texture-induced effective metric $g_{\tau ,\mathrm{ij}}^{\text{eff}}\left(r\right)$. (**B** and **D**): potential lensing from the emergent scalar potential $V_{\tau }\left(r\right)$. Potential lensing produces opposite deflections of the two τ sectors (focusing/defocusing). In contrast, metric lensing generates a transmission asymmetry.

The deflection of the radial component is negligible, $\Delta p_{r,\tau }≃0$. This result shows, once more, that there is no deflection along the crystal axes, $\varphi_{0}=0,\pi /2$, and that it depends on the τ sector. In addition, we also see that the deflection gets negligible when $v_{r}$ becomes sizable. This corresponds to exactly the limit where effects of the quantum metric are of relevance. We verified that this behavior is also qualitatively found when solving the semiclassical equations numerically for finite *w*, see Fig. 5 (B and D).

Let us continue with (ii) and consider the metric-induced lensing effects for the same radial domain wall example. In this case, the texture-induced quantum metric is $g_{\mathrm{ij}}\left(r\right)=\phi^{\prime }{\left(r\right)}^{2}{\hat{r}}_{i}{\hat{r}}_{j}/4$ and the effective metric that the electron feel is $g_{\text{eff},\tau }^{\mathrm{ij}}\left(r\right)=K_{\tau }^{\mathrm{ij}}-\beta \left(r\right){\left(K_{\tau }\hat{r}\right)}^{i}{\left(K_{\tau }\hat{r}\right)}^{j}$ with $\beta \left(r\right)=\left(\hbar^{2}/2J\right)\phi^{\prime }{\left(r\right)}^{2}.$ The velocity, $\dot{r}$, of an itinerant electron is determined by the conjugate momentum, Π, through the Hamilton equation $\dot{r}=2g_{\text{eff},\tau }\left(r\right)\Pi$. Projecting this equation onto the radial direction, $\hat{r}$, gives

$$\dot{r}=2v_{g,r}^{\left(0\right)}\left(\varphi \right)\left[1-\beta \left(r\right){\hat{r}}^{T}K_{\tau }\hat{r}\right]\equiv 2v_{g,r}^{\left(0\right)}Z_{\tau }\left(r,\varphi \right)$$ (15)

where $v_{g,r}^{\left(0\right)}={\hat{r}}^{T}K_{\tau }\Pi$ is the radial component of the group velocity of the uniform altermagnet and $Z_{\tau }\left(r,\varphi \right)$ is a τ-dependent transmission factor. It is defined through the second relation in Eq. 15 and can be explicitly written as $Z_{\tau }\left(r,\varphi \right)=1-\beta \left(r\right)\left(\varrho_{0}+\tau \varrho_{z}\text{cos}2\varphi \right)$. This finding has interesting consequences by considering three different regimes.

If $Z_{\tau }>0$, then the velocity $\dot{r}$ has the same sign as $v_{g,r}^{\left(0\right)}$. Hence, the ray will move in the direction of the radial momentum projection. If $Z_{\tau }=0$, then $\dot{r}=0$ and the ray will be tangent to the wall. If $Z_{\tau }<0$, then $\dot{r}$ has opposite sign as $v_{g,r}^{\left(0\right)}$. Hence, the ray would be strongly redirected by the domain wall. Because of the τ dependence of $Z_{\tau }$, a special situation could be approached when say, $Z_{+}>0$ but $Z_{-}<0$. In this situation, the semiclassical viewpoint suggests that trajectories can be strongly spin dependent, which we illustrate numerically in Fig. 5 (A and C). In terms of the microscopic system parameters, the condition $Z_{+}>0$ but $Z_{-}<0$ corresponds, at the level of the semiclassical considerations, to ${\left(\varrho_{0}-\varrho_{z}\text{cos}2\varphi \right)}^{-1}<\beta <{\left(\varrho_{0}+\varrho_{z}\text{cos}2\varphi \right)}^{-1}.$ We emphasize that both spin-dependent scattering effects discussed in this section would be absent in the antiferromagnetic limit ($\varrho_{z}\to 0$) where neither $Z_{\tau }$ nor $V_{\tau }$ depends on τ (also note that $\Delta p_{\varphi ,\tau }\propto \varrho_{z}$ in Eq. 14).

### Emergent spin-orbit coupling and odd-parity magnetism

We lastly allow for a finite $t_{x}$ such that there is an additional, nontrivial second-order process: The transverse gauge-field components $A^{⊥}$ change the spin of the electron and, subsequently, the $t_{x}$ term swaps the sublattice, bringing us back to the low-energy subspace (with S=−1). This time, however, the component of the spin has changed, leading to an additional contribution $\Delta {\hat{H}}_{\text{rot}}^{\left(1\right)}$ to ${\hat{H}}_{\text{rot}}^{\left(1\right)}$, which involves $\sigma_{x,y}$. While the full expression can be found in the Supplementary Materials, the correction reads in the semiclassical limit as

$$\Delta H_{\text{rot}}=g_{p}\left(r\right)\cdot \sigma_{\phi \left(r\right)}$$ (16A)

with “emergent spin-orbit field”

$$g_{p}=g_{0}\;p_{j}\left[\begin{array}{c} \text{sin}\left(\theta \right)\partial_{j}\phi \\ \partial_{j}\theta \end{array}\right],\;g_{0}=-\frac{\hbar \varrho_{0}t_{x}}{J}$$ (16B)

and rotated Pauli matrices $\sigma_{\phi }=R_{\phi }{\left(\sigma_{x},\sigma_{y}\right)}^{T}$ where $R_{\phi }$ rotates two-component vectors by an angle ϕ. Note that the third second-order process, based on changing the sublattice twice with $t_{x}$, does not lead to a nontrivial operator in the low-energy subspace and, hence, will not be discussed here.

It holds $g_{p}=-g_{-p}$ such that the term in Eq. 1 preserves time-reversal symmetry but is odd under inversion (at fixed position r). Hence, it can locally be thought of as effective spin-orbit coupling. However, this term does not arise from relativistic corrections but, instead, from nontrivial magnetic textures. Consequently, one might also think of Eq. 16, A and B, as representing some form of spontaneous generation of spin-orbit coupling (*54*–*56*) or an admixed odd-parity (anti-alter)magnetic component (*32*, *57*). We emphasize, however, that Eq. 16B is not unique to altermagnets as it persists in the limit $\varrho_{z}\to 0$, as opposed to the lensing effects of the previous section.

**Fig. 6.**
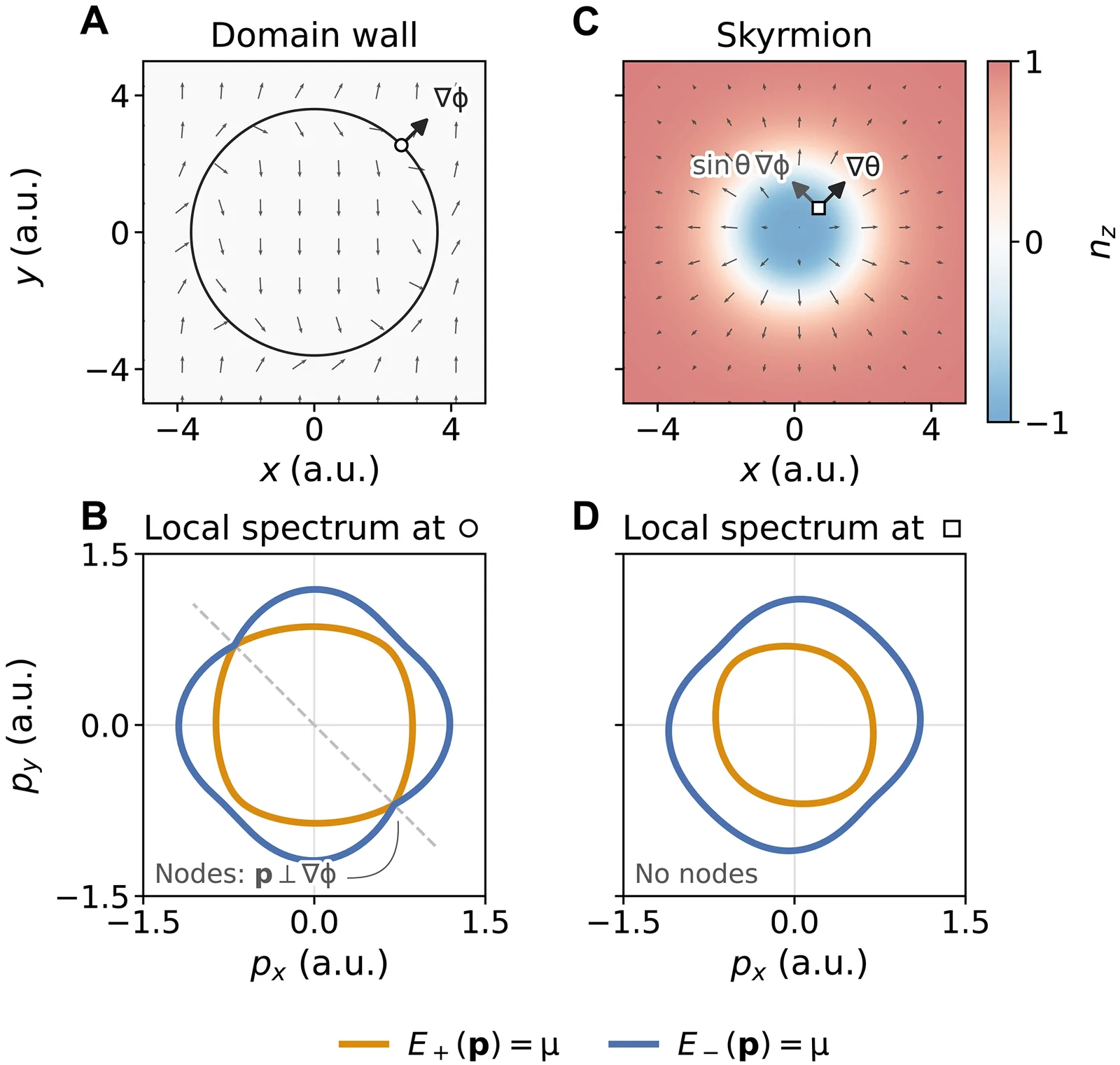
Emergent spin-orbit coupling and odd-parity magnetism from altermagnetic textures. (**A**) Circular Néel domain wall. Faint arrows show the in-plane Néel vector field, n(r). The marked point lies at azimuthal angle φ=π/4, where the emergent Zeeman field, $V_{z}\propto \text{cos}2\varphi$, vanishes. (**B**) Local constant-energy contours, $E_{\pm }\left(p\right)=\mu$, evaluated at that point. Because *V_z_* = 0, the splitting arises purely from the metric-induced spin-orbit term, $g_{p}\propto p\cdot \nabla \phi$. The splitting vanishes for p⊥∇ϕ (dashed line), producing nodes. (**C**) Skyrmion texture. The color scale shows $n_{z}$. Arrows indicate the in-plane components of n(r). (**D**) Local constant-energy contours at the point in (C). Here, both ∇θ and sinθ∇ϕ contribute to $g_{p}$, which eliminates any nodes.

To discuss concrete examples, let us begin with coplanar textures, e.g., by setting θ=π/2 but allowing for ϕ=ϕ(r), which includes our domain wall example in Fig. 1 as a special case. From Eq. 16B, we find $g_{p}/g_{0}={\left(p\cdot \nabla \phi ,0\right)}^{T}$. As can be read off from the corresponding local spectrum

$$E_{\pm }\left(p\right)=\varepsilon_{0,p}+\varrho_{0}V_{0}\pm \sqrt{{\left(t_{z,p}+\varrho_{z}V_{z}\right)}^{2}+g_{p}^{2}}$$ (17)

of our effective, semiclassical 2×2 low-energy Hamiltonian $H_{\text{rot}}^{\left(\text{eff}\right)}$ (where we neglected the nontrivial metric in Eq. 11 for simplicity), the spin-orbit splitting is $|g_{p}|$ and thus finite except for ***p*** perpendicular to ∇ϕ. In Fig. 6 (A and B), we illustrate this behavior for a domain wall, where $\nabla \phi =\hat{r}\phi^{\prime }\left(r\right)$, by focusing on a single point at the boundary (indicated in Fig. 6B). Note that a planar spiral, i.e., ϕ(r)=q⋅r, leads to the spatially constant spin-orbit field, $g_{p}={\left(p\cdot q,0\right)}^{T}$, of the same form and, thus, the same type of splitting everywhere in space. We emphasize the similarity to the vanishing of the spin splitting in one direction in the *p*-wave magnets discussed in (*57*).

Spatially varying altermagnets can also induce spin-orbit splittings without nodal directions in $g_{p}$. This, however, necessitates that sin(θ)∇ϕ and ∇θ are linearly independent, which in turn is equivalent to having a noncoplanar n(r). For instance, $\phi =q_{1}\cdot r$ and $\theta =q_{2}\cdot r$ yield a finite $|g_{p}|$ for all momenta p≠0 as long as $q_{1}$ and $q_{2}$ are linearly independent and for points in space with $\text{sin}q_{2}\cdot r\neq 0$. Another important noncoplanar structure is a skyrmion, see Fig. 6C, which also gives rise to extended regions in space with non-nodal spin-orbit splitting, see Fig. 6D.

We lastly point out that including an additional, subleading term in the intersublattice hopping, $t_{x,p}=t_{x}+\varrho_{x}p^{2}$, already generates a term of the same form as Eq. 16, A and B, at order $1/J^{0}$; now with $g_{0}=\hbar \varrho_{3}$. Which one is more relevant depends on how large *J* is compared to $t_{x}\varrho_{0}/\varrho_{x}$.

Starting from the four-band model in Eq. 1, with a slowly varying altermagnetic order parameter n(r,t), we have derived an effective two-band model describing the electronic spectrum in a rotating reference frame and studied the physical consequences of the different corrections that emerge relative to the homogeneous limit $n\left(r,t\right)=n_{0}$. First, to leading (zeroth) order 1/J, we find that noncoplanar textures induce the emergent magnetic and electric fields given in Eq. 3, which are opposite for the two sublattices and persist in the antiferromagnetic limit ($t_{z}\to 0$). We also uncover an emergent Zeeman field $V_{z}$, which, in contrast, is unique to the altermagnet. It involves the quantum metric $g_{\mathrm{ij}}$ of the magnetic texture and as such does not require noncoplanar order, as well as the momentum-space structure of the altermagnetic order parameter (via $\eta^{\mathrm{ij}}$ and $T^{\text{ijkl}}$ in Eqs. 5 and 7 for a *d*-wave and *g*-wave altermagnet, respectively). Since the associated local spin polarization can be probed experimentally, e.g., with scanning SQUIDs or NV magnetometry, this not only allows to distinguish altermagnets from antiferromagnets but also to access the predominant angular dependence of the altermagnetic order parameter (cf. Fig. 2, A and B). We have further shown that the emergent Zeeman field also leads to a spin-dependent lensing effect at inhomogeneities such as domain walls, see Fig. 5 (B and D).

At order 1/J, additional corrections appear, which we divide into two categories. The first type preserves the spin/sublattice degree of freedom and can be thought of as an inhomogeneous, anisotropic contribution to the effective mass tensor or emulating curved space for electronic motion; as with the Zeeman field, it is unique to the altermagnet, involves the metric $g_{\mathrm{ij}}$ and the form of the altermagnetic order parameter, and also introduces a spin-dependent lensing and, depending on parameters, spin-dependent transmissions, see Fig. 5 (A and C). The second type of corrections to order 1/J mixes the spin species and can be interpreted as emergent spin-orbit coupling or the local admixture of an effectively odd-parity magnetic component. It can further gap out the nodes of the altermagnetic spin splitting of the bands.

Together, we have demonstrated that textures in altermagnets lead to particularly rich electronic properties. On the one hand, our findings show that taking into account such inhomogeneities, which are inevitable in experimental samples, is imperative for a proper understanding of the local and global electronic properties. On the other hand, altermagnetic textures also provide unprecedented opportunities for locally engineering electronic properties of interest, emulating dynamics in curved space and analog gravity, creating spin-dependent electronic lenses and filters, and probing the altermagnetic order parameter symmetry experimentally. Note that at the final stages of the preparation of this manuscript, (*58*) appeared, which also discusses the impact of altermagnetic textures on electronic properties, albeit with a different focus.

## MATERIALS AND METHODS

This theoretical study is based on the minimal four-band Hamiltonian in Eq. 1. We transform to a local rotating spin frame, project to the low-energy S=−1 sector, and include virtual transitions to the high-energy sector through a Schrieffer-Wolff transformation up to first order in 1/J. The effective Hamiltonian obtained in this way is used to derive the emergent electromagnetic fields, geometric potentials, kinetic energy corrections, and spin-orbit terms discussed in the main text. Lensing trajectories are calculated in a semiclassical approximation by solving Hamilton’s equations of motion. The predicted scanning SQUID flux signal is obtained from the Biot-Savart law applied to the local current density, which is computed from the conductivity tensor within a relaxation time approximation. The detailed derivations and parameters used are provided in the Supplementary Materials.

## Supplementary Materials

### This PDF file includes:

Supplementary Text S1 to S7 Tables S1 to S5 References
