# Supplementary material for: Altermagnetic spin textures: Emergent electrodynamics, quantum geometry, and probes
Source: Sci Adv. 2026 Sep 18;12(38):eaeh1411. doi: 10.1126/sciadv.aeh1411 (PMC13588161; doi:10.1126/sciadv.aeh1411)
Supplement: Supplementary file 1 [file sciadv.aeh1411_sm.pdf]

Science Advances
AAAS

Supplementary Materials for

Altermagnetic spin textures: Emergent electrodynamics, quantum geometry, and probes

Constantin Schrade and Mathias S. Scheurer

Corresponding author: Constantin Schrade, cschrade@lsu.edu;
Mathias S. Scheurer, mathias.scheurer@itp3.uni-stuttgart.de

Sci. Adv. **12**, eaeh1411 (2026)
DOI: 10.1126/sciadv.aeh1411

This PDF file includes:

Supplementary Text S1 to S7
Tables S1 to S5
References

S1. ROTATING FRAME HAMILTONIAN

In this section, we provide more details on the rotating frame Hamiltonian in which the quantization axis on each sublattice follows the local magnetization. For concreteness, we will focus on the case of a time-independent altermagnetic texture.

As a *first step*, we redisplay (for convenience) the Hamiltonian for the altermagnetic spin texture,

$$\hat{\mathcal{H}} = \hat{\varepsilon}_{0,\hat{\boldsymbol{p}}}\tau_0 + \hat{t}_{x,\hat{\boldsymbol{p}}}\tau_x + \hat{t}_{z,\hat{\boldsymbol{p}}}\tau_z + J\tau_z\,\boldsymbol{n}(\boldsymbol{r})\cdot\boldsymbol{s}. \tag{S1}$$

as introduced in Eq. (1) of the main text. We then parameterize the unit vector field $\boldsymbol{n}(\boldsymbol{r})$ as,

$$\boldsymbol{n}(\boldsymbol{r}) = \left(\sin\theta(\boldsymbol{r})\cos\phi(\boldsymbol{r}), \sin\theta(\boldsymbol{r})\sin\phi(\boldsymbol{r}), \cos\theta(\boldsymbol{r})\right), \tag{S2}$$

and introduce a position-dependent spin rotation that aligns $\boldsymbol{n}(\boldsymbol{r})$ with the spin quantization axis,

$$U(\boldsymbol{r}) = \exp\left[-\frac{i}{2}\,\theta(\boldsymbol{r})\left(-\sin\phi(\boldsymbol{r})s_x + \cos\phi(\boldsymbol{r})s_y\right)\right], \qquad U^\dagger(\boldsymbol{r})\left(\boldsymbol{s}\cdot\boldsymbol{n}(\boldsymbol{r})\right)U(\boldsymbol{r}) = s_z. \tag{S3}$$

As a *second step*, we define the rotating-frame Hamiltonian as,

$$\hat{\mathcal{H}}_{\mathrm{rot}} \equiv U^\dagger(\boldsymbol{r})\,\hat{\mathcal{H}}\,U(\boldsymbol{r}). \tag{S4}$$

which evaluates to,

$$\hat{\mathcal{H}}_{\mathrm{rot}} = \hat{\varepsilon}_{0,\hat{\boldsymbol{\pi}}}\tau_0 + \hat{t}_{x,\hat{\boldsymbol{\pi}}}\tau_x + \hat{t}_{z,\hat{\boldsymbol{\pi}}}\tau_z + J\tau_z s_z, \qquad \hat{\boldsymbol{\pi}} = U^\dagger(\boldsymbol{r})\,\hat{\boldsymbol{p}}\,U(\boldsymbol{r}) = \hat{\boldsymbol{p}} - \boldsymbol{A}(\boldsymbol{r}). \tag{S5}$$

We note that the spatial variation of the altermagnetic texture gives rise to emergent SU(2) gauge fields that couple to the electron momentum,

$$A_i(\boldsymbol{r}) = i\hbar U^\dagger(\boldsymbol{r})\partial_i U(\boldsymbol{r}), \qquad \boldsymbol{A}(\boldsymbol{r}) = (A_x(\boldsymbol{r}), A_y(\boldsymbol{r}), A_z(\boldsymbol{r})). \tag{S6}$$

For the parameterization introduced above, these SU(2) gauge fields take the explicit form,

As a *third step*, it is useful to separate the SU(2) gauge fields into components that are parallel and perpendicular to the quantization axis,

$$A_i(\boldsymbol{r}) = A_i^{\parallel}(\boldsymbol{r}) + A_i^{\perp}(\boldsymbol{r}), \qquad A_i^{\parallel} \propto s_z, \qquad A_i^{\perp} \propto s_x, s_y, \tag{S7}$$

and thus commute and anticommute with $\tau_z s_z$, respectively. These components are explicitly given by

$$\begin{aligned} A_i^{\parallel}(\boldsymbol{r}) &= -\hbar\,\partial_i\phi(\boldsymbol{r})\sin^2\left(\frac{\theta(\boldsymbol{r})}{2}\right)s_z \\ A_i^{\perp}(\boldsymbol{r}) &= \frac{\hbar}{2}\left[\left(\partial_i\theta(\boldsymbol{r})\right)\boldsymbol{e}_\phi(\boldsymbol{r}) - \sin\theta(\boldsymbol{r})\left(\partial_i\phi(\boldsymbol{r})\right)\boldsymbol{e}_r(\boldsymbol{r})\right]\cdot(s_x, s_y), \end{aligned} \tag{S8}$$

with $\boldsymbol{e}_r(\boldsymbol{r}) = (\cos\phi(\boldsymbol{r}), \sin\phi(\boldsymbol{r}))^T$ and $\boldsymbol{e}_\phi(\boldsymbol{r}) = (-\sin\phi(\boldsymbol{r}), \cos\phi(\boldsymbol{r}))^T$.

Using this decomposition, we also introduce a momentum operator shifted by the longitudinal SU(2) gauge field,

$$\hat{\Pi}_i = \hat{p}_i - A_i^{\parallel}, \tag{S9}$$

so that the we can write

$$\hat{\pi}_i = \hat{\Pi}_i - A_i^{\perp}. \tag{S10}$$

This formulation will be the starting point for our subsequent derivation of the low-energy effective Hamiltonian.

S2. EFFECTIVE HAMILTONIAN

In this section, we provide details on the derivations of the effective Hamiltonians shown in the main text.

S2.1. Schrieffer-Wolff Transformation

We begin by redisplaying (for convenience) the rotating-frame Hamiltonian given in Eq. (2) of the main text,

$$\hat{\mathcal{H}}_{\text{rot}} = \hat{\varepsilon}_{0,\hat{\boldsymbol{\pi}}}\tau_0 + \hat{t}_{x,\hat{\boldsymbol{\pi}}}\tau_x + \hat{t}_{z,\hat{\boldsymbol{\pi}}}\tau_z + JS \tag{S11}$$

We then introduce the operator,

$$S \equiv \tau_z s_z, \tag{S12}$$

whose eigenvalues $s = \pm 1$ label the two subspaces separated by the large energy scale, J. In the rotating frame, S is a good quantum number for distinguishing the low-energy ($s = -1$) and high-energy ($s = +1$) subspaces. The projection operators onto these subspaces are given by,

$$P_{\pm} = \frac{1}{2}(1 \pm S). \tag{S13}$$

It will now be convenient to decompose $\hat{\mathcal{H}}_{\text{rot}}$ into diagonal and off-diagonal blocks with respect to the low- and high-energy subspaces,

$$H_{1,2} \equiv P_{\mp}\hat{\mathcal{H}}_{\text{rot}}P_{\mp}, \qquad \Lambda \equiv P_{-}\hat{\mathcal{H}}_{\text{rot}}P_{+}, \tag{S14}$$

With these definitions, we find that the low-energy effective Schrieffer-Wolff Hamiltonian is (to first order in $1/J$) given by,

$$H_{\text{eff}} = H_1 - \frac{1}{2J}\Lambda\Lambda^{\dagger} \tag{S15}$$

We will now evaluate this effective Hamiltonian for the d-wave and g-wave altermagnet.

S2.2. Effective Hamiltonian for the d-wave altermagnet (0th order in 1/J)

For our first example of the d-wave altermagnet, we have $\hat{\varepsilon}_{0,\hat{\boldsymbol{p}}} = \varrho_0(\hat{p}_x^2 + \hat{p}_y^2)$, $\hat{t}_{x,\hat{\boldsymbol{p}}} = t_x$, and $\hat{t}_{z,\hat{\boldsymbol{p}}} = \varrho_z(\hat{p}_x^2 - \hat{p}_y^2)$. In this case, the kinetic term in our texture Hamiltonian can be written compactly as,

$$\hat{\varepsilon}_{0,\hat{\boldsymbol{\pi}}}\tau_0 + \hat{t}_{z,\hat{\boldsymbol{\pi}}}\tau_z = \hat{\pi}_i K^{ij}\hat{\pi}_j, \quad \text{with} \quad K^{ij} = \varrho_0\delta^{ij} + \varrho_z\tau_z\eta^{ij} \quad \text{and} \quad \eta = \text{diag}(1,-1). \tag{S16}$$

We initially decompose the SU(2) gauge fields as $\hat{\pi}_i = \hat{\Pi}_i - A_i^{\perp}$ with $\hat{\Pi}_i = \hat{k}_i - A_i^{\parallel}$ and rewrite the first contribution to the kinetic term as,

$$\begin{aligned}
\hat{\pi}_i K^{ij}\hat{\pi}_j &= (\hat{\Pi}_i - A_i^{\perp})K^{ij}(\hat{\Pi}_j - A_j^{\perp}) \\
&= \hat{\Pi}_i K^{ij}\hat{\Pi}_j - (\hat{\Pi}_i K^{ij}A_j^{\perp} + A_i^{\perp}K^{ij}\hat{\Pi}_j) + A_i^{\perp}K^{ij}A_j^{\perp} \\
&= \hat{\Pi}_i K^{ij}\hat{\Pi}_j - (\hat{\Pi}_i K^{ij}A_j^{\perp} + K^{ij}A_j^{\perp}\hat{\Pi}_i) + A_i^{\perp}K^{ij}A_j^{\perp} \\
&= \hat{\Pi}_i K^{ij}\hat{\Pi}_j - \{\hat{\Pi}_i,\ K^{ij}A_j^{\perp}\} + A_i^{\perp}K^{ij}A_j^{\perp},
\end{aligned} \tag{S17}$$

Using this result, the full rotating-frame Hamiltonian takes on the form,

$$H_{\text{rot}} = \left[\hat{\Pi}_i K^{ij}\hat{\Pi}_j + A_i^{\perp}K^{ij}A_j^{\perp}\right] - \{\hat{\Pi}_i,\ K^{ij}A_j^{\perp}\} + t_x\tau_x + JS. \tag{S18}$$

With this rewriting, we are now in the position to evaluate the lowest-order contribution to our effective Hamiltonian,

$$\begin{aligned} H^{(0)}_{\rm rot} &= H_1 \\ &= -J + P_-[\hat{\Pi}_i K^{ij}\hat{\Pi}_j + A^\perp_i K^{ij} A^\perp_j]P_- \\ &= -J + P_-[\varrho_0(\hat{\Pi}_x^2+\hat{\Pi}_y^2) + \varrho_z\tau_z(\hat{\Pi}_x^2-\hat{\Pi}_y^2) + \big(\varrho_0 V_1 + \varrho_z\tau_z V_z\big)]P_-, \end{aligned} \tag{S19}$$

where we have defined the quantities,

$$\begin{aligned} V_0 &= \frac{\hbar^2}{4}[(\partial_x\theta)^2 + (\partial_y\theta)^2 + \sin^2\theta\big((\partial_x\phi)^2+(\partial_y\phi)^2\big)] = \hbar^2\delta^{ij}\, g_{ij}(\boldsymbol{r}), \\ V_z &= \frac{\hbar^2}{4}[(\partial_x\theta)^2 - (\partial_y\theta)^2 + \sin^2\theta\big((\partial_x\phi)^2-(\partial_y\phi)^2\big)] = \hbar^2\eta^{ij}\, g_{ij}(\boldsymbol{r}), \end{aligned} \tag{S20}$$

with $\eta = \mathrm{diag}(1,-1)$ and the quantum metric $g_{ij}(\boldsymbol{r}) = \partial_i\boldsymbol{n}(\boldsymbol{r})\cdot\partial_j\boldsymbol{n}(\boldsymbol{r})/4$. Introducing the Pauli matrices σ_j that act in the low-energy subspace, with basis ($\tau=+, s=\downarrow$) and ($\tau=-, s=\uparrow$), we obtain the effective low-energy Hamiltonian given by (upon dropping an irrelevant constant)

$$H^{(0)}_{\rm rot} = \varrho_0\sigma_0(\hat{\Pi}_x^2+\hat{\Pi}_y^2) + \varrho_z\sigma_z(\hat{\Pi}_x^2-\hat{\Pi}_y^2) + \big(\varrho_0 V_1 + \varrho_z\sigma_z V_z\big). \tag{S21}$$

This concludes our derivation for the effective Hamiltonian of the d-wave altermagnet system.

S2.3. Effective Hamiltonian for the g-wave altermagnet ($0^{\rm th}$ order in 1/J)

For our second example of the g-wave altermagnet, we again set $\hat{\varepsilon}_{0,\hat{\boldsymbol{p}}} = \varrho_0(\hat{p}_x^2+\hat{p}_y^2)$, $\hat{t}_{x,\hat{\boldsymbol{p}}} = t_x$. Moreover, we conveniently write,

$$\hat{t}_{z,\hat{\boldsymbol{\pi}}} = \bar{\varrho}_z\mathcal{W}[\hat{\pi}_x\hat{\pi}_y(\hat{\pi}_x^2-\hat{\pi}_y^2)] = \bar{\varrho}_z T^{ijkl}\mathcal{W}[\hat{\pi}_i\hat{\pi}_j\hat{\pi}_k\hat{\pi}_l]. \tag{S22}$$

where we have defined the totally symmetric rank-4 tensor T^{ijkl} as,

$$T^{ijkl} = \frac{1}{4!}\frac{1}{\bar{\varrho}_z}\left.\frac{\partial^4 t_{z,\boldsymbol{p}}}{\partial p^i\partial p^j\partial p^k\partial p^l}\right|_{\boldsymbol{p}=0}. \tag{S23}$$

In particular, we remark that its non-zero components are given by,

$$T^{xxxy} = T^{xxyx} = T^{xyxx} = T^{yxxx} = \frac{1}{4}, \qquad T^{xyyy} = T^{yxyy} = T^{yyxy} = T^{yyyx} = -\frac{1}{4}, \tag{S24}$$

As a next step, we insert our decomposition of the shifted momenta in terms of the longitudinal and transversal SU(2) gauge fields, $\hat{\pi}_i = \hat{\Pi}_i - \hat{A}^\perp_i$ and $\hat{\Pi}_i = \hat{k}_i - \hat{A}^\parallel_i$. Projected onto the $S=-1$ subspace, we find that

$$P_-\hat{t}_{z,\hat{\boldsymbol{\pi}}}P_- = \bar{\varrho}_z P_- T^{ijkl}\left[\mathcal{W}[\hat{\Pi}_i\hat{\Pi}_j\hat{\Pi}_k\hat{\Pi}_l] + 6\,\mathcal{W}[\hat{\Pi}_i\hat{\Pi}_j\hat{A}^\perp_k\hat{A}^\perp_l] + \mathcal{W}[\hat{A}^\perp_i\hat{A}^\perp_j\hat{A}^\perp_k\hat{A}^\perp_l]\right]P_- \tag{S25}$$

where we have used that only products involving an even number of transversal gauge field factors are non-zero upon projecting with P_-.

To further simplify the above expression, we note that

$$\begin{aligned} \hat{A}^\perp_k\hat{A}^\perp_l &= \frac{1}{2}\{\hat{A}^\perp_k,\hat{A}^\perp_l\} + \frac{1}{2}[\hat{A}^\perp_k,\hat{A}^\perp_l] \\ &= \hbar^2(g_{ij} + i\,\chi_{ij}\,s_z) \end{aligned} \tag{S26}$$

where $\chi_{ij} = \boldsymbol{n}\cdot(\partial_i\boldsymbol{n}\times\partial_j\boldsymbol{n})/4$ is the scalar spin chirality. This result can be seen from the explicit form of the transverse gauge potential, $\hat{A}^\perp_i = \frac{\hbar}{2}((\partial_i\theta)\mathbf{e}_\phi - \sin\theta\,(\partial_i\phi)\mathbf{e}_r)\cdot(s_x,s_y)$, and the quantum metric, $g_{jk}(\mathbf{r}) = [(\partial_j\theta)(\partial_k\theta) + \sin^2\theta\,(\partial_j\phi)(\partial_k\phi)]/4 = (\partial_i\boldsymbol{n})\cdot(\partial_j\boldsymbol{n})/4$.

We can now write the second term in our expansion of $P_-\hat{t}_{z,\hat{\boldsymbol{\pi}}}P_-$ as,

$$
\begin{aligned}
T^{ijkl}\mathcal{W}[\hat{\Pi}_i\hat{\Pi}_j\hat{A}_k^\perp\hat{A}_l^\perp] &= \frac{1}{2}T^{ijkl}\mathcal{W}[\hat{\Pi}_i\hat{\Pi}_j[\hat{A}_k^\perp,\hat{A}_l^\perp]] + \frac{1}{2}T^{ijkl}\mathcal{W}[\hat{\Pi}_i\hat{\Pi}_j\{\hat{A}_k^\perp,\hat{A}_l^\perp\}] \\
&= \frac{1}{2}T^{ijkl}\mathcal{W}[\hat{\Pi}_i\hat{\Pi}_j\{\hat{A}_k^\perp,\hat{A}_l^\perp\}] \\
&= \hbar^2 T^{ijkl}\mathcal{W}[\hat{\Pi}_i\hat{\Pi}_j\, g_{kl}(\mathbf{r})].
\end{aligned} \tag{S27}
$$

Here, we have noted that T^{ijkl} and $\{\hat{A}_k^\perp,\hat{A}_l^\perp\}$ are both even under $k\leftrightarrow l$, while $[\hat{A}_k^\perp,\hat{A}_l^\perp]$ is odd under $k\leftrightarrow l$ and thus vanishes upon carrying out the summation.

In a similar way, we find for the last term in the expansion of $P_-\hat{t}_{z,\hat{\boldsymbol{\pi}}}P_-$ that,

$$
T^{ijkl}\hat{A}_i^\perp\hat{A}_j^\perp\hat{A}_k^\perp\hat{A}_l^\perp = \hbar^4 T^{ijkl}[(g_{ij}g_{kl}-\chi_{ij}\chi_{kl}) + i(g_{ij}\chi_{kl}+\chi_{ij}g_{kl})s_z] = \hbar^4 T^{ijkl}g_{ij}g_{kl}, \tag{S28}
$$

where the last equality follows because T^{ijkl} is totally symmetric and χ_{ij} is antisymmetric under $i\leftrightarrow j$.

We can now combine these results to arrive at,

$$
\hat{\mathcal{H}}_{\rm rot}^{(0)} = P_-[\hat{\varepsilon}_{0,\hat{\boldsymbol{\Pi}}}\tau_0 + (\hat{t}_{z,\hat{\boldsymbol{\Pi}}} + \delta\hat{t}_{z,\hat{\boldsymbol{\Pi}}})\tau_z + \varrho_0 V_0\tau_0 + \bar{\varrho}_z V_z\tau_z]P_-, \tag{S29}
$$

with

$$
\begin{aligned}
\delta\hat{t}_{z,\hat{\mathbf{p}}} &= 6\hbar^2\bar{\varrho}_z T^{ijkl}\mathcal{W}[\hat{p}_i\hat{p}_j g_{kl}(\boldsymbol{r},t)] \\
V_z &= \hbar^4 T^{ijkl}g_{ij}(\boldsymbol{r},t)g_{kl}(\boldsymbol{r},t),
\end{aligned} \tag{S30}
$$

This concludes our derivation of the effective Hamiltonian to $0^{\rm th}$ order in $1/J$ for the g-wave altermagnet example.

S2.4. Effective Hamiltonian for the d-wave altermagnet ($1^{\rm st}$ order in 1/J)

As a next step, we return to our first example of the d-wave altermagnet and compute the correction to the effective Hamiltonian in $1^{\rm st}$ order in $1/J$.

For this purpose, it is initially useful to formulate a few definitions and identities. The *first identity* is

$$
A_i^\perp A_j^\perp = \hbar^2(g_{ij}+i\chi_{ij}s_z) \quad \text{with} \quad \{A_i^\perp,A_j^\perp\} = 2\hbar^2 g_{ij} \quad \text{and} \quad [A_i^\perp,A_j^\perp] = 2i\hbar^2\chi_{ij}s_z \tag{S31}
$$

with $\chi_{ij} = \boldsymbol{n}\cdot(\partial_i\boldsymbol{n}\times\partial_j\boldsymbol{n})/4$. The *second identity* is

$$
g_{ij} = \sum_{a=x,y} e_{ia}e_{ja}, \tag{S32}
$$

where,

$$
\begin{aligned}
A_i^\perp = \hbar\sum_{a=x,y} e_{ia}s_a, \quad \text{with} \quad e_{ix} &= -\frac{1}{2}\left[(\partial_i\theta)\sin\phi + \sin\theta(\partial_i\phi)\cos\phi\right], \\
e_{iy} &= \frac{1}{2}\left[(\partial_i\theta)(\cos\phi) - \sin\theta(\partial_i\phi)\sin\phi\right].
\end{aligned} \tag{S33}
$$

The *third identity* is,

$$
\{\hat{\Pi}_i, A_j^\perp\} = \{\hat{p}_i, A_j^\perp\} = \hbar\sum_{a=x,y}\{\hat{p}_i, e_{ja}\}s_a \tag{S34}
$$

where we used that $\{s_z, A_j^\perp\} = 0$ in the first equality.

With the help of these identities, we can now compute the 1st order correction in $1/J$ to the effective Hamiltonian. For this purpose, we write the off-diagonal blocks as,

$$\Lambda = P_- H_{\rm rot} P_+ = P_-\big(t_x\tau_x - \{\hat{\Pi}_i,\ K^{ij}A_j^{\perp}\}\big)P_+ \tag{S35}$$

and define,

$$\Lambda = \Lambda_\Delta + \Lambda_\pi, \qquad \Lambda_\Delta \equiv P_-(t_x\tau_x)P_+, \qquad \Lambda_\pi \equiv -P_-\{\hat{\Pi}_i,\ K^{ij}A_j^{\perp}\}P_+ \tag{S36}$$

We then decompose the following product,

$$\Lambda\Lambda^\dagger = \Lambda_\Delta\Lambda_\Delta^\dagger + \Lambda_\pi\Lambda_\pi^\dagger + \Lambda_\Delta\Lambda_\pi^\dagger + \Lambda_\pi\Lambda_\Delta^\dagger. \tag{S37}$$

as it appears in our effective Hamiltonian and evaluate this expression term by term:

First, we consider the contribution originating from the sublattice hybridization. This term takes the form,

$$\Lambda_\Delta\Lambda_\Delta^\dagger = t_x^2 P_-, \tag{S38}$$

which gives a constant energy shift within the low-energy ($S = -1$) subspace.

Second, we consider the product $\Lambda_\pi\Lambda_\pi^\dagger$. Here, we first write,

$$\begin{aligned}\Lambda_\pi &= -P_-\{\hat{\Pi}_i, K^{ij}A_j^{\perp}\}P_+ = -\hbar K^{ij}\sum_{a=x,y}\{\hat{p}_i, e_{ja}\}(P_- s_a P_+)\\ \Lambda_\pi^\dagger &= -\hbar K^{k\ell}\sum_{a'=x,y}\{\hat{p}_k, e_{\ell a'}\}\,(P_+ s_{a'} P_-).\end{aligned} \tag{S39}$$

We then find that the product of these operators can be written as,

$$\begin{aligned}\Lambda_\pi\Lambda_\pi^\dagger &= \hbar^2 K^{ij}K^{k\ell}\sum_{a,a'=x,y}\{\hat{p}_i, e_{ja}\}\{\hat{p}_k, e_{\ell a'}\}\,(P_- s_a P_+ s_{a'} P_-)\\ &= \hbar^2 K^{ij}K^{k\ell}\sum_{a,a'=x,y}\{\hat{p}_i, e_{ja}\}\{\hat{p}_k, e_{\ell a'}\}P_-(s_a s_{a'})P_-\\ &= \hbar^2 K^{ij}K^{k\ell}\left[\sum_{a=x,y}\{\hat{p}_i, e_{ja}\}\{\hat{p}_k, e_{\ell a}\}\,P_- + i\sum_{a,a'=x,y}\varepsilon_{aa'}\{\hat{p}_i, e_{ja}\}\{\hat{p}_k, e_{\ell a'}\}\,(P_- s_z P_-)\right]\end{aligned} \tag{S40}$$

where we have used that $P_- s_a P_+ s_{a'} P_- = P_- s_a s_{a'} P_-$ and, for $a, a' = x, y$, we have $s_a s_{a'} = \delta_{aa'} + i\varepsilon_{aa'} s_z$ with the totally antisymmetric tensor $\varepsilon_{aa'}$ that satisfies $\varepsilon_{xy} = 1$.

In the semiclassical limit, we find for the first term in the above expression,

$$\Lambda_\pi\Lambda_\pi^\dagger \to 4\hbar^2(p_i K^{ij})\,g_{j\ell}(K^{\ell m}p_m). \tag{S41}$$

Finally, we evaluate the cross terms involving both Λ_Δ and Λ_π.

$$\Lambda_\Delta\Lambda_\pi^\dagger + \Lambda_\pi\Lambda_\Delta^\dagger = -t_x P_-\left\{\tau_x, \{\hat{\Pi}_i, K^{ij}A_j^{\perp}\}\right\}P_-. \tag{S42}$$

where we have used that for any pair of operators, A and B, that anti-commute with S, we have $(P_- A P_+)(P_+ B P_-) + (P_- B P_+)(P_+ A P_-) = P_-\{A, B\}P_-$ with $O_1 = t_x\tau_x$ and $O_2 = -\{\hat{\Pi}_i,\ K^{ij}A_j^{\perp}\}$.

To further simplify this, we note the general anti-commutator identity $\{A, \{B, C\}\} = \{B, \{A, C\}\}$ if $[A, B] = 0$. Using this identity combined with $[\tau_x, \hat{\Pi}_i] = 0$ and $[\tau_x, A_j^{\perp}] = 0$ (since $\hat{\Pi}_i$ and $A_j^{\perp}$ are comprised of $s_{x,y,z}$ Pauli

matrices), we find that,

$$
\begin{aligned}
\Lambda_\Delta \Lambda_\pi^\dagger + \Lambda_\pi \Lambda_\Delta^\dagger &= -t_x P_- \left\{\tau_x, \{\hat{\Pi}_i, K^{ij} A_j^\perp\}\right\} P_- \\
&= -t_x P_- \left\{\hat{\Pi}_i, \{\tau_x, K^{ij} A_j^\perp\}\right\} P_- \\
&= -t_x P_- \left\{\hat{\Pi}_i, \{\tau_x, K^{ij}\} A_j^\perp\right\} P_- \\
&= -t_x P_- \left\{\hat{\Pi}_i, \{\tau_x, K^{ij}\} A_j^\perp\right\} \\
&= -t_x P_- \left\{\hat{\Pi}_i, \{\tau_x, K^{ij}\} A_j^\perp\right\} P_- \\
&= -2\varrho_0 t_x \sum_{i=x,y} P_- \{\hat{\Pi}_i, \tau_x A_i^\perp\} P_- \\
&= -2\varrho_0 t_x \sum_{i=x,y} P_- \tau_x \{\hat{\Pi}_i, A_i^\perp\} P_- \\
&= -2\varrho_0 t_x \sum_{i=x,y} P_- \tau_x \{\hat{p}, A_i^\perp\} P_- \\
&= -2\hbar\varrho_0 t_x \sum_{i=x,y} \sum_{a=x,y} \{\hat{\Pi}_i, e_{ia}(\boldsymbol{r})\} P_- (\tau_x s_a) P_-
\end{aligned}
\tag{S43}
$$

Here, we also used $\{A_i^\parallel, A_i^\perp\} = 0$ and $\{\tau_x, K^{ij}\} = \{\tau_x, \varrho_0 \delta^{ij} + \varrho_z \tau_z \eta^{ij}\} = 2\varrho_0 \delta^{ij} \tau_x$. In summary, with this, we see that the full quantum mechanical correction is given by,

$$
\begin{aligned}
\hat{\mathcal{H}}^{(1)}_{\rm rot} = -\frac{1}{2J}\Big[& t_x^2 P_- + \hbar^2 K^{ij} K^{k\ell} \Big(\sum_{a=x,y} \{\hat{p}_i, e_{ja}(\boldsymbol{r})\}\{\hat{p}_k, e_{\ell a}(\boldsymbol{r})\} P_- + i \sum_{a,a'=x,y} \varepsilon_{aa'} \{\hat{p}_i, e_{ja}(\boldsymbol{r}\}\{\hat{p}_k, e_{\ell a'}(\boldsymbol{r})\} \left(P_- s_z P_-\right)\Big) \\
& - 2\hbar\varrho_0 t_x \sum_{i=x,y} \sum_{a=x,y} \{\hat{p}_i, e_{ia}(\boldsymbol{r})\} P_- (\tau_x s_a) P_- \Big]
\end{aligned}
\tag{S44}
$$

We will now discuss the term $\propto \varrho_0 t_x$ in more detail.

Defining, as before, Pauli matrices σ_j acting in the low-energy subspace (P_- projects on) with basis choice ($\tau = +, s =\downarrow$) and ($\tau = -, s =\uparrow$), the associated contribution to the Hamiltonian reads as

$$
\Delta\hat{\mathcal{H}}_{\rm rot} = \frac{\hbar\varrho_0 t_x}{J} \sum_{i=x,y} \left(\{\hat{p}_i, e_{ix}(\boldsymbol{r})\}\sigma_x - \{\hat{p}_i, e_{iy}(\boldsymbol{r})\}\sigma_y\right), \tag{S45}
$$

where we explicitly indicated that $e_{ia}(\boldsymbol{r})$, as defined in Eq. (S33), depend in general on position. Again in the semi-classical limit, we can effectively replace $\{\hat{p}_i, e_{ia}(\boldsymbol{r})\} \to 2p_i e_{ia}$ leading to

$$
\Delta\mathcal{H}_{\rm rot} = \frac{2\hbar\varrho_0 t_x}{J} \sum_{i=x,y} p_i \left(e_{ix}(\boldsymbol{r})\sigma_x - e_{iy}(\boldsymbol{r})\sigma_y\right) = \mathfrak{g}_{\boldsymbol{p}}(\boldsymbol{r}) \cdot \boldsymbol{\sigma}_{\phi(\boldsymbol{r})}, \tag{S46}
$$

where we introduced the spatially varying spin-orbit field and rotated Pauli matrices given by (suppressing position dependence for notational simplicity)

$$
\mathfrak{g}_{\boldsymbol{p}} = -\frac{\hbar\varrho_0 t_x}{J} \begin{pmatrix} \sin(\theta)\, \boldsymbol{p}\cdot\boldsymbol{\nabla}\phi \\ \boldsymbol{p}\cdot\boldsymbol{\nabla}\theta \end{pmatrix}, \quad \text{and} \quad \boldsymbol{\sigma}_\phi = \mathcal{R}_\phi \begin{pmatrix} \sigma_x \\ \sigma_y \end{pmatrix}, \quad \mathcal{R}_\phi = \begin{pmatrix} \cos\phi & -\sin\phi \\ \sin\phi & \cos\phi \end{pmatrix}, \tag{S47}
$$

respectively.

To derive the condition stated in the main text, we first note that

$$
\mathfrak{g}_{\boldsymbol{p}}^2 = \frac{\hbar^2 \varrho_0^2 t_x^2}{J^2} \left[\sin^2\theta (\boldsymbol{p}\cdot\boldsymbol{\nabla}\phi)^2 + (\boldsymbol{p}\cdot\boldsymbol{\nabla}\theta)^2\right] \tag{S48}
$$

is non-vanishing for all $\boldsymbol{p} \neq 0$ if and only if $\sin\theta \neq 0$ and the two vectors $\boldsymbol{\nabla}\phi$ and $\boldsymbol{\nabla}\theta$ are linearly independent. At the same time, the texture is (locally) non-collinear if and only if

$$
\boldsymbol{n}\cdot(\partial_x \boldsymbol{n} \times \partial_y \boldsymbol{n}) = \sin\theta \left[(\partial_x\theta)(\partial_y\phi) - (\partial_y\theta)(\partial_x\phi)\right] \tag{S49}
$$

$$= \sin\theta \det[\boldsymbol{\nabla}\theta\ \boldsymbol{\nabla}\phi] \neq 0 \quad \Leftrightarrow \quad \sin\theta \neq 0 \text{ and } \boldsymbol{\nabla}\phi,\ \boldsymbol{\nabla}\theta \text{ linearly independent.} \tag{S50}$$

Finally, we point out that extending the interlayer term to depend on momentum via $t_{x,\boldsymbol{p}} = t_x + \varrho_x \boldsymbol{p}^2$ already induces a term that couples ($\tau = +, s =\downarrow$) and ($\tau = -, s =\uparrow$) on the level of the first term in Eq. (S15). This leads to the additional contribution to Eq. (S21) given by

$$\Delta H = -\varrho_3 \sum_{i=x,y} \left(\{\hat{p}_i, e_{ix}(\boldsymbol{r})\}\sigma_x - \{\hat{p}_i, e_{iy}(\boldsymbol{r})\}\sigma_y\right), \tag{S51}$$

which is identical to Eq. (S45) except for the different prefactor.

S3. LENSING

In this section, we provide more details on the derivation of the lensing effects of an incoming semiclassical ray from the potential term.

As a starting point, we define a circular domain wall texture as

$$\theta(\boldsymbol{r}) = \frac{\pi}{2}, \qquad \phi(\boldsymbol{r}) = \phi(r) = \frac{\pi}{2}\tanh\left(\frac{r-R}{w}\right), \qquad \boldsymbol{n}(r,\varphi) = (\cos\phi(r), \sin\phi(r), 0). \tag{S52}$$

where w is the domain wall width and R is its radius. We note that,

$$\boldsymbol{n} \cdot (\partial_x \boldsymbol{n} \times \partial_y \boldsymbol{n}) = 0 \tag{S53}$$

for this texture and, as a result, the orbital magnetic field contribution vanishes.

We want to evaluate the potentials, V_z and V_0, for this domain wall texture. For this purpose, we first need to compute the metric,

$$g_{ij} = \frac{1}{4}(\partial_i\phi)(\partial_j\phi) \quad \text{so that} \quad g_{xx} = \frac{1}{4}\phi'(r)^2\cos^2\varphi, \quad \text{and} \quad g_{yy} = \frac{1}{4}\phi'(r)^2\sin^2\varphi. \tag{S54}$$

From this form of the metric, we find that,

$$\begin{aligned} V_z(\boldsymbol{r}) &= \hbar^2\eta^{ij}g_{ij} = \hbar^2(g_{xx} - g_{yy}) = \frac{\hbar^2}{4}\phi'(r)^2\cos 2\varphi = f(r)\cos 2\varphi \quad \text{with} \quad f(r) = \frac{\hbar^2}{4}\phi'(r)^2 \\ V_0(\boldsymbol{r}) &= \hbar^2\delta^{ij}g_{ij} = \hbar^2(g_{xx} + g_{yy}) = \frac{\hbar^2}{4}\phi'(r)^2 \end{aligned} \tag{S55}$$

As a next step, we write down the semiclassical equations of motion,

$$\dot{\boldsymbol{r}} = \frac{1}{\hbar}\nabla_{\boldsymbol{k}}\varepsilon_{\pm}, \quad \text{and} \quad \hbar\dot{\boldsymbol{k}} = -\nabla_{\boldsymbol{r}}\varepsilon_{\pm}. \tag{S56}$$

where we have defined the energies,

$$\varepsilon_\tau(\boldsymbol{k}, \boldsymbol{r}) = \varepsilon_0(\boldsymbol{k}) + \tau t_z(\boldsymbol{k}) + \varrho_0 V_0(\boldsymbol{r}) + \tau\varrho_z V_z(\boldsymbol{r}). \tag{S57}$$

We will primarily be interested in the equation of motion,

$$\hbar\dot{\boldsymbol{k}} = -\hat{\boldsymbol{r}}[\varrho_0 f'(r) \pm \varrho_z f'(r)\cos 2\varphi] \pm \hat{\boldsymbol{\varphi}}\varrho_z\frac{2f(r)}{r}\sin 2\varphi \tag{S58}$$

to understand the change in momentum of an incoming ray due to the domain wall. Here, we have defined the unit vectors in the radial and angular directions $\hat{\boldsymbol{r}} = (\cos\varphi, \sin\varphi)$ and $\hat{\boldsymbol{\varphi}} = (-\sin\varphi, \cos\varphi)$. Moreover, we have used the polar representation of the gradient, $\nabla_{\boldsymbol{r}} = \hat{\boldsymbol{r}}\partial_r + \hat{\boldsymbol{\varphi}}\frac{1}{r}\partial_\varphi$. Lastly, it will be useful to separately write down the components of this equation in the radial and angular directions,

$$\hbar\dot{k}_r = -[\varrho_0 f'(r) \pm \varrho_z f'(r)\cos 2\varphi] \quad \text{and} \quad \hbar\dot{k}_\varphi = \pm\varrho_z\frac{2f(r)}{r}\sin 2\varphi \tag{S59}$$

Let us now assume that we have some incoming ray with velocity,

$$\boldsymbol{v}_{\mathrm{in},\tau} \simeq \frac{1}{\hbar}\nabla_{\boldsymbol{k}}[\varepsilon_0(\boldsymbol{k}) + \tau t_z(\boldsymbol{k})]\big|_{\boldsymbol{k}=\boldsymbol{k}_{\mathrm{in}}}. \tag{S60}$$

The ray hits the domain wall at,

$$\boldsymbol{r}_0 = R\hat{\boldsymbol{r}}(\varphi_0), \tag{S61}$$

which defines an angle, φ_0. The velocity components at this point of impact are,

$$v_{r,\tau} = \boldsymbol{v}_{\mathrm{in},\tau}\cdot\hat{\boldsymbol{r}}(\varphi_0) \quad \text{and} \quad v_{\varphi,\tau} = \boldsymbol{v}_{\mathrm{in},\tau}\cdot\hat{\boldsymbol{\varphi}}(\varphi_0). \tag{S62}$$

We assume now that the domain wall, i.e., the region where the potentials V_0 and V_z are finite, is very thin, $w \ll R$. In this case, we will assume that $\varphi(t)$ and $r(t)$ are unchanged for the moment that the ray transverses through the domain wall,

$$\varphi_\tau(t) \simeq \varphi_0, \quad r_\tau(t) \simeq R, \quad dt \simeq \frac{dr}{v_{r,\tau}}. \tag{S63}$$

We then first compute the change in the angular component of the momentum by integrating the semiclassical equation in the angular direction,

$$\begin{aligned}
\Delta k_{\varphi,\pm} &= \pm\frac{\varrho_z}{\hbar}\int dt \frac{2f(r(t))}{r(t)}\sin 2\varphi(t) \\
&\simeq \pm\frac{\varrho_z}{\hbar}\frac{2\sin 2\varphi_0}{R}\frac{1}{v_{r,\pm}}\int_{-\infty}^{\infty} dr f(r) \\
&= \pm\frac{\varrho_z}{\hbar}\frac{2\sin 2\varphi_0}{R}\frac{1}{v_{r,\pm}}\left(\frac{\hbar^2\pi^2}{12w}\right) \\
&= \pm\frac{\hbar\varrho_z\pi^2}{6wR}\frac{\sin 2\varphi_0}{v_{r,\pm}}.
\end{aligned} \tag{S64}$$

We can perform a similar calculation for the radial component of the momentum,

$$\begin{aligned}
\Delta k_{r,\pm} &= \int_{-\infty}^{\infty} dt \dot{k}_r = -\frac{1}{\hbar}\int_{-\infty}^{\infty} dt[\varrho_0 f'(r(t)) \pm \varrho_z f'(r(t))\cos 2\varphi(t)] \\
&\simeq -\frac{1}{\hbar v_{r,\pm}}[\varrho_0 \pm \varrho_z\cos 2\varphi_0]\int_{-\infty}^{\infty} dr f'(r) \\
&= -\frac{1}{\hbar v_{r,\pm}}[\varrho_0 \pm \varrho_z\cos 2\varphi_0][f(\infty) - f(-\infty)] \\
&= 0,
\end{aligned} \tag{S65}$$

where in the last equality we have used that $f(\infty) = f(-\infty) \simeq 0$.

Hence, we see that the ray experiences primarily a momentum deflection along the angular coordinate.

S4. ESTIMATION OF THE SIZE OF THE EMERGENT ZEEMAN FIELD

In this section, we estimate the size of the emergent Zeeman field in candidate altermagnets.

A first example is Mn_5Si_3, which has recently been discussed as a candidate d-wave altermagnet *(59–62)*. We consider a planar radial domain wall with radius R and width w. The emergent Zeeman term at the center of the wall is $|V_z(R)| = \hbar^2\pi^2/16w^2$. The corresponding emergent Zeeman energy is therefore $E_z \equiv \varrho_z|V_z(R)| = \varrho_z\hbar^2\pi^2/16w^2$. To estimate the coefficient ϱ_z, we relate it to the reported altermagnetic spin-splitting scale. We use $\varrho_z\hbar^2 \sim \Delta_{\mathrm{AM}}/2k_{\mathrm{BZ}}^2$,

Wall width w	Zeeman energy $E_z = \varrho_z \lvert V_z(R)\rvert$	Equivalent field $B_{\rm eq} = E_z/\mu_B$	Flux signal Φ
4 nm	104 μeV	1.80 T	4.1 $\mu\Phi_0$
10 nm	16.7 μeV	289 mT	1.6 $\mu\Phi_0$
20 nm	4.18 μeV	72.2 mT	0.82 $\mu\Phi_0$
50 nm	0.668 μeV	11.5 mT	0.33 $\mu\Phi_0$

TABLE S1. **Estimated emergent Zeeman fields and scanning-SQUID signals for Mn_5Si_3.** Estimates are shown for a planar circular domain wall with width w. The second column gives the emergent Zeeman energy at the domain wall radius, R, as $E_z = \varrho_z|V_z(R)|$. The third column gives the equivalent magnetic field strength, $B_{\rm eq} = E_z/\mu_B$. The last column gives the estimated flux through a scanning-SQUID pickup loop. The parameters used for these estimates are given in the text.

with $\Delta_{\rm AM} = 150$ meV *(28)*. As a momentum scale, we take $k_{\rm BZ} \equiv |\Gamma M| = 2\pi/\sqrt{3}a$, where $a \sim 6.9$ Å is the lattice constant *(62)*. This gives the estimate $E_z(w) \sim 16.7\ \mu\text{eV}\ (10\ \text{nm}/w)^2$. It is useful to express the same energy as an equivalent Zeeman field, $B_{\rm eq} \equiv E_z/\mu_B$, where μ_B is the Bohr magneton. Because $E_z \propto 1/w^2$, the estimate depends strongly on the domain-wall width, as summarized in Table S1. For example, for $w = 10$ nm, we obtain $B_{\rm eq} \sim 289$ mT.

A second example is MnTe, which has been considered as a candidate g-wave altermagnet *(63, 64)*. We use $\Delta_{\rm AM} = 0.8$ eV at $k_{\rm BZ} \sim 0.43|\Gamma K|$, with $|\Gamma K| = 4\pi/3a$ and $a \sim 4.158$ Å *(64)*. This gives $\bar{\varrho}_z\hbar^4 \sim \Delta_{\rm AM}/2k_{\rm BZ}^4 \sim 11.4$ eV Å^4. For a circular wall, the corresponding g-wave Zeeman scale is $E_z(w) \equiv \bar{\varrho}_z|V_z(R)| = \bar{\varrho}_z\hbar^4\pi^4/1024w^4$, or $E_z(w) \sim 108\ \mu\text{eV}\ (1\ \text{nm}/w)^4$. The equivalent field is $B_{\rm eq}(w) \sim 1.87\ \text{T}\ (1\ \text{nm}/w)^4$. Because this contribution scales as $1/w^4$, MnTe is most favorable in the sharp-wall regime. For example, a wall width $w = 5$ nm gives $E_z \sim 0.173\ \mu$eV and $B_{\rm eq} \sim 3.0$ mT. We remark that atomically sharp domain walls have already been observed in certain antiferromagnets *(65)*.

S5. MEASURING THE EMERGENT ZEEMAN FIELDS WITH A SCANNING SQUID

In this sections, we provide more details on the measurement of the emergent Zeeman fields of altermagentic texture with a scanning superconducting quantum interference device (SQUID).

S5.1. Conductivity tensor

As first step, we will show that the conductivity tensor of our altermagnetic texture system acquires a non-uniformity that is determined by the emergent Zeeman field, $V_z(\boldsymbol{r})$. More specifically, the conductivity tensor is of the form,

$$\sigma_{ij}(\boldsymbol{r}) = \chi_0\delta_{ij} - \chi_0' V_0(\boldsymbol{r})\delta_{ij} - \chi_z V_z(\boldsymbol{r})\eta_{ij}. \tag{S66}$$

To compute the coefficients $(\chi_0, \chi_0', \chi_z)$ and verify this form of the conductivity tensor, we compute the Drude conductivity in the zero-temperature limit through the formula,

$$\sigma_{ij}(\boldsymbol{r}) = e^2\tau_{\rm rel}\sum_{\tau=\pm}\int\frac{d^2p}{(2\pi\hbar)^2}v_{i,\tau}v_{j,\tau}\,\delta\left(\mu - \varepsilon_\tau(\boldsymbol{p},\boldsymbol{r})\right), \tag{S67}$$

where τ is the relaxation time and the local energy dispersion and group velocities are given by,

$$\varepsilon_\tau(\boldsymbol{p},\boldsymbol{r}) = \kappa_{x,\tau}p_x^2 + \kappa_{y,\tau}p_y^2 + U_\tau(\boldsymbol{r}) \quad \text{and} \quad v_{x,\tau} = 2\kappa_{x,\tau}p_x, \quad v_{y,\tau} = 2\kappa_{y,\tau}p_y. \tag{S68}$$

Here, we have adopted the following short-hand notation for the coefficients of the kinetic terms and the potential term,

$$\kappa_{x,\tau} = \varrho_0 + \tau\varrho_z, \quad \kappa_{y,\tau} = \varrho_0 - \tau\varrho_z \quad \text{and} \quad U_\tau(\boldsymbol{r}) = \varrho_0 V_0(\boldsymbol{r}) + \tau\varrho_z V_z(\boldsymbol{r}). \tag{S69}$$

With these definitions, we find that the σ_{xx} component of the conductivity tensor is,

$$\begin{aligned}
\sigma_{xx} &= e^2\tau_{\text{rel}} \sum_{\tau=\pm} \int \frac{d^2p}{(2\pi\hbar)^2} (2\kappa_{x,\tau}p_x)^2 \delta\left(\mu - U_\tau - \kappa_{x,\tau}p_x^2 - \kappa_{y,\tau}p_y^2\right) \\
&= e^2\tau_{\text{rel}} \sum_{\tau=\pm} \frac{4\kappa_{x,\tau}}{\sqrt{\kappa_{x,\tau}\kappa_{y,\tau}}} \int \frac{d^2q}{(2\pi\hbar)^2} q_x^2\, \delta(\mu - U_\tau - q^2) \\
&= e^2\tau_{\text{rel}} \sum_{\tau=\pm} \frac{4\kappa_{x,\tau}}{\sqrt{\kappa_{x,\tau}\kappa_{y,\tau}}} \int_0^{2\pi} d\theta\, \cos^2\theta \int_0^\infty dq\, q^3\, \delta(\mu - U_\tau - q^2) \\
&= \frac{e^2\tau_{\text{rel}}}{2\pi\hbar^2} \sum_{\tau=\pm} \frac{\kappa_{x,\tau}}{\sqrt{\kappa_{x,\tau}\kappa_{y,\tau}}}(\mu - U_\tau) \\
&= \frac{e^2\tau_{\text{rel}}}{2\pi\hbar^2} \frac{1}{\sqrt{\varrho_0^2 - \varrho_z^2}} \sum_{\tau=\pm} (\varrho_0 + \tau\varrho_z)(\mu - \varrho_0 V_0 - \tau\varrho_z V_z) \\
&= \frac{e^2\tau_{\text{rel}}}{\pi\hbar^2\sqrt{\varrho_0^2 - \varrho_z^2}}(\varrho_0\mu - \varrho_0^2 V_0 - \varrho_z^2 V_z)
\end{aligned} \tag{S70}$$

where we have employed the substitution, $q_x = p_x\sqrt{\kappa_{x,\tau}}$ and $q_y = p_y\sqrt{\kappa_{y,\tau}}$ in the second equality. In the third equality, we have used polar coordinates, $q_x = q\cos\theta$.

We similarly find for the σ_{xx} component of the conductivity tensor,

$$\begin{aligned}
\sigma_{yy} &= \frac{e^2\tau}{2\pi\hbar^2} \frac{1}{\sqrt{\varrho_0^2 - \varrho_z^2}} \sum_{\tau=\pm} (\varrho_0 - \tau\varrho_z)(\mu - \varrho_0 V_0 - \tau\varrho_z V_z) \\
&= \frac{e^2\tau_{\text{rel}}}{\pi\hbar^2\sqrt{\varrho_0^2 - \varrho_z^2}}(\varrho_0\mu - \varrho_0^2 V_0 + \varrho_z^2 V_z).
\end{aligned} \tag{S71}$$

The off-diagonal components, σ_{xy} and σ_{xy}, vanish, because they involve an integration of a function that is odd-in-momentum.

From these results, we can read off the form of the coefficients of the previously proposed form of the conductivity tensor,

$$\chi_0 = \frac{e^2\tau_{\text{rel}}}{\pi\hbar^2}\frac{\varrho_0\mu}{\sqrt{\varrho_0^2 - \varrho_z^2}}, \quad \chi_0' = \frac{e^2\tau_{\text{rel}}}{\pi\hbar^2}\frac{\varrho_0^2}{\sqrt{\varrho_0^2 - \varrho_z^2}}, \quad \chi_z = \frac{e^2\tau_{\text{rel}}}{\pi\hbar^2}\frac{\varrho_z^2}{\sqrt{\varrho_0^2 - \varrho_z^2}}. \tag{S72}$$

For the subsequent calculations, it will be useful to separate the conductivity tensor into a contribution that is not due to the texture and one that arises from the texture,

$$\sigma_{ij}(\boldsymbol{r}) = \sigma_0\delta_{ij} + \delta\sigma_{ij}(\boldsymbol{r}) \tag{S73}$$

with

$$\delta\sigma_{xx}(\boldsymbol{r}) = -\chi_0' V_0(\boldsymbol{r}) - \chi_z V_z(\boldsymbol{r}) \quad \text{and} \quad \delta\sigma_{yy}(\boldsymbol{r}) = -\chi_0' V_0(\boldsymbol{r}) + \chi_z V_z(\boldsymbol{r}). \tag{S74}$$

From this rewriting, we see that $V_0(\boldsymbol{r})$ changes the conductivity in the same way along x and y. In comparison, $V_z(\boldsymbol{r})$ changes the conductivity with opposite sign along x and y.

S5.2. Current

We now consider an electric field that is applied along the x-direction. Then the resulting current in the sample is,

$$\boldsymbol{j}^{(x)}(\boldsymbol{r}) = \sigma(\boldsymbol{r})\boldsymbol{E}^{(x)}(\boldsymbol{r}) \quad \text{with} \quad \boldsymbol{E}^{(x)}(\boldsymbol{r}) = E_x\hat{x} - \nabla\phi_x(\boldsymbol{r}), \tag{S75}$$

where E_x is the magnitude of the externally applied electric field and $\phi_x(\boldsymbol{r})$ is an internally generated scalar potential that we will choose appropriately to satisfy current conservation. We remark that this additional scalar potential

arises due to the position-dependence of the conductivity tensor and would vanish if $\delta\sigma_{xx}(\boldsymbol{r}) = 0$. As a result, it is *at least* of linear order in $\delta\sigma_{xx}(\boldsymbol{r})$.

Using the above form of the conductivity tensor, we find that the current is given by,

$$\boldsymbol{j}^{(x)}(\boldsymbol{r}) \simeq \sigma_0 E_x \hat{x} + E_x \delta\sigma_{xx}(\boldsymbol{r})\hat{x} - \sigma_0 \nabla\phi_x(\boldsymbol{r}) \tag{S76}$$

Here, we have retained only terms that are *at most* of linear order in $\delta\sigma_{xx}(\boldsymbol{r})$. In particular, we have dropped the term $\propto \delta\sigma_{xx}(\boldsymbol{r})\nabla\phi_x(\boldsymbol{r})$, which is at least quadratic in $\delta\sigma_{xx}(\boldsymbol{r})$. This approximation is justified if the texture varies sufficiently slowly, so that the potentials, $V_0(\boldsymbol{r})$ and $V_z(\boldsymbol{r})$, can be considered as perturbations.

The internally generated scalar potential is determined from current conservation, $\nabla \cdot \boldsymbol{j}^{(x)} = 0$. Using the expression above for the current, this constraint is equivalent to,

$$\sigma_0 \nabla^2 \phi_x(\boldsymbol{r}) = E_x \partial_x \delta\sigma_{xx}(\boldsymbol{r}). \tag{S77}$$

In momentum-space, this constraint can be solved,

$$\phi_x(\boldsymbol{q}) = -\frac{iE_x q_x}{\sigma_0 q^2}\delta\sigma_{xx}(\boldsymbol{q}). \tag{S78}$$

The Fourier components of the current are then given by,

$$\begin{aligned} \boldsymbol{j}^{(x)}(\boldsymbol{q}) &= \sigma_0 E_x (2\pi)^2 \delta(\boldsymbol{q})\,\hat{x} + E_x \delta\sigma_{xx}(\boldsymbol{q})\hat{x} - \sigma_0 i\boldsymbol{q}\phi_x(\boldsymbol{q}) \\ &= \sigma_0 E_x (2\pi)^2 \delta(\boldsymbol{q})\hat{x} + E_x \left[\hat{x} - \frac{q_x}{q^2}\boldsymbol{q}\right]\delta\sigma_{xx}(\boldsymbol{q}). \end{aligned} \tag{S79}$$

If an electric field, E_y, is applied along the y-direction, we can obtain a corresponding expression for the current,

$$\boldsymbol{j}^{(y)}(\boldsymbol{q}) = \sigma_0 E_y (2\pi)^2 \delta(\boldsymbol{q})\hat{y} + E_y \left[\hat{y} - \frac{q_y}{q^2}\boldsymbol{q}\right]\delta\sigma_{yy}(\boldsymbol{q}). \tag{S80}$$

S5.3. Vector potential generated by the current

We next want to derive the vector potential that is generated by the current density that flows in the x-y-plane of the system,

$$\boldsymbol{J}(\boldsymbol{r}, z) = \boldsymbol{j}(\boldsymbol{r})\delta(z). \tag{S81}$$

The vector potential associated with this current density is determined by,

$$\nabla^2 \boldsymbol{A} = -\mu_0 \boldsymbol{J}, \tag{S82}$$

We will, henceforth, work in a gauge with $A_z = 0$.

To solve the differential equation for the vector potential, we perform a Fourier transform in the x-y-plane,

$$(\partial_z^2 - q^2)\boldsymbol{A}(\boldsymbol{q}, z) = -\mu_0 \boldsymbol{j}(\boldsymbol{q})\delta(z). \tag{S83}$$

The solution for this equation when $z \neq 0$ and $q > 0$ is given by,

$$\boldsymbol{A}(\boldsymbol{q}, z) = \boldsymbol{A}_0(\boldsymbol{q})e^{-q|z|}. \tag{S84}$$

The coefficient, $\boldsymbol{A}_0(\boldsymbol{q})$, is determined from the boundary conditions. Specifically, we integrate the differential equation across $z = 0$ and find the constraint,

$$\partial_z \boldsymbol{A}(\boldsymbol{q}, 0^+) - \partial_z \boldsymbol{A}(\boldsymbol{q}, 0^-) = -\mu_0 \boldsymbol{j}(\boldsymbol{q}). \tag{S85}$$

This constraint determines,

$$\boldsymbol{A}_0(\boldsymbol{q}) = \frac{\mu_0}{2q}\boldsymbol{j}(\boldsymbol{q}) \quad \text{and, hence,} \quad \boldsymbol{A}(\boldsymbol{q}, z) = \frac{\mu_0}{2q}\boldsymbol{j}(\boldsymbol{q})e^{-q|z|}. \tag{S86}$$

We remark that when $z \neq 0$ and $q = 0$ the solution is given by,

$$\boldsymbol{A}(\boldsymbol{0}, z) = -\frac{\mu_0}{2}\mathbf{j}(\mathbf{0})|z|, \tag{S87}$$

which satisfies $\partial_z^2 \boldsymbol{A}(\boldsymbol{0}, z) = 0$ and the boundary conditions.

S5.4. Magnetic field generated by the current

As a next step, we will compute the magnetic field along the z-direction from the vector potential.

The magnetic field at a height, $h > 0$, in the z-direction is then given by, $B_z(\boldsymbol{r}, h) = \partial_x A_y(\boldsymbol{r}, h) - \partial_y A_x(\boldsymbol{r}, h)$, or in Fourier space, $B_z(\boldsymbol{q}, h) = iq_x A_y(\boldsymbol{q}, h) - iq_y A_x(\boldsymbol{q}, h)$. We thus see that the $\boldsymbol{q} = 0$ does not contribute the magnetic field along the z-direction. Henceforth, we will thus assume that $\boldsymbol{q} \neq 0$.

Inserting our result for the vector potential into the expression for the magnetic field gives,

$$\begin{aligned} B_z(\boldsymbol{q}, h) &= \frac{\mu_0}{2} e^{-qh} \frac{i(q_x j_y(\boldsymbol{q}) - q_y j_x(\boldsymbol{q}))}{q} \\ &= \frac{\mu_0}{2} e^{-qh} \frac{i(\hat{z} \cdot (\boldsymbol{q} \times \boldsymbol{j}(\boldsymbol{q})))}{q} \end{aligned} \tag{S88}$$

For the first case of the previous section when the current density is along the x-direction, $\boldsymbol{j}^{(x)}(\boldsymbol{r})$, we find that the resulting magnetic field is,

$$\begin{aligned} B_z^{(x)}(\boldsymbol{q}, h) &= -\frac{\mu_0}{2} e^{-qh} \frac{iq_y}{q} E_x \, \delta\sigma_{xx}(\boldsymbol{q}) \\ &= \frac{\mu_0}{2} e^{-qh} \frac{iq_y}{q} E_x \left[\chi_0' V_0(\boldsymbol{q}) + \chi_z V_z(\boldsymbol{q})\right]. \end{aligned} \tag{S89}$$

Similarly, for the second case when the current density is along the y-direction, $\boldsymbol{j}^{(y)}(\boldsymbol{r})$, we find that the resulting magnetic field is,

$$\begin{aligned} B_z^{(y)}(\boldsymbol{q}, h) &= \frac{\mu_0}{2} e^{-qh} \frac{iq_x}{q} E_y \, \delta\sigma_{yy}(\boldsymbol{q}) \\ &= \frac{\mu_0}{2} e^{-qh} \frac{iq_x}{q} E_y \left[-\chi_0' V_0(\boldsymbol{q}) + \chi_z V_z(\boldsymbol{q})\right]. \end{aligned} \tag{S90}$$

S5.5. Flux measurement and extracting the emergent Zeeman field

In a scanning SQUID experiment, the measured quantity is the magnetic flux through a pick-up loop,

$$\Phi^{(x/y)}(\boldsymbol{R}) = \int d^2r \, P(\boldsymbol{R} - \boldsymbol{r}) B_z^{(x/y)}(\boldsymbol{r}, h). \tag{S91}$$

Here, $\boldsymbol{R}$ is the scan coordinate and $P(\boldsymbol{R} - \boldsymbol{r})$ is the pickup-loop point spread function *(66)*. In Fourier space, we thus have,

$$\Phi^{(x/y)}(\boldsymbol{q}, h) = P(\mathbf{q}) B_z^{(x/y)}(\boldsymbol{q}, h). \tag{S92}$$

For the two considered current directions, the flux profile in momentum space is then given by,

$$\begin{aligned} \Phi^{(x)}(\boldsymbol{q}, h) &= \frac{\mu_0}{2} P(\boldsymbol{q}) e^{-qh} \frac{iq_y}{q} E_x \left[\chi_0' V_0(\boldsymbol{q}) + \chi_z V_z(\boldsymbol{q})\right], \\ \Phi^{(y)}(\boldsymbol{q}, h) &= \frac{\mu_0}{2} P(\boldsymbol{q}) e^{-qh} \frac{iq_x}{q} E_y \left[-\chi_0' V_0(\boldsymbol{q}) + \chi_z V_z(\boldsymbol{q})\right]. \end{aligned} \tag{S93}$$

The momentum-space representations of the scalar potentials are then given by,

$$\begin{aligned} V_z(\boldsymbol{q}) &= \frac{e^{qh}}{\mu_0 \chi_z P(\boldsymbol{q})} \left[\frac{q}{iq_y} \frac{\Phi^{(x)}(\boldsymbol{q}, h)}{E_x} + \frac{q}{iq_x} \frac{\Phi^{(y)}(\boldsymbol{q}, h)}{E_y}\right] \\ V_0(\boldsymbol{q}) &= \frac{e^{qh}}{\mu_0 \chi_0' P(\boldsymbol{q})} \left[\frac{q}{iq_y} \frac{\Phi^{(x)}(\boldsymbol{q}, h)}{E_x} - \frac{q}{iq_x} \frac{\Phi^{(y)}(\boldsymbol{q}, h)}{E_y}\right]. \end{aligned} \tag{S94}$$

The position-space representation is then obtained through,

$$V_z(\boldsymbol{r}) = \int \frac{d^2q}{(2\pi)^2} e^{i\boldsymbol{q}\cdot\boldsymbol{r}} V_z(\boldsymbol{q}). \tag{S95}$$

These results point to the following algorithm for measuring the scalar potential:

1. Measure two scanning SQUID flux maps, one with current bias along x and one along y.
2. Take the Fourier transform of both maps.
3. Compute the scalar potentials, $V_z(\boldsymbol{q})$ and $V_0(\boldsymbol{q})$, from the above expressions.
4. Inverse Fourier transform to obtain $V_z(\boldsymbol{r})$ and $V_0(\boldsymbol{r})$.

S5.6. Estimate of the strength of the flux signal

Motivated by this protocol, we estimate the flux scale seen by a scanning SQUID. We consider a sample of width W carrying a total current I. Far from the domain wall, the sheet current is $j_0 = I/W$. Near the wall, the emergent Zeeman term changes the local conductivity anisotropically. This produces a local current modulation of order $\delta j/j_0 \sim \chi_z V_z(R)/\chi_0$. Using the Drude coefficients, this becomes $\delta j/j_0 \sim (\varrho_z/\varrho_0)(E_z/\mu)$, where $E_z = \varrho_z|V_z(R)|$ and μ is the Fermi-energy scale. A wall segment of width w carries an excess or deficit sheet current of at most $\delta I \sim w\delta j$. At a height h above the sample, this produces a real stray field of order $B_z \sim \mu_0 \delta I (2\pi h)^{-1}$. The SQUID converts this field into a flux $\Phi \sim A_{\text{eff}} B_z$, where A_{eff} is the effective pickup-loop area.

We now apply this estimate to Mn_5Si_3. We take $\varrho_z/\varrho_0 \sim 0.15$ as the relative size of the altermagnetic splitting. The carrier density reported for Mn_5Si_3 thin films is of order $n \sim 10^{22}$ cm^{-3} *(59)*. A free-electron estimate then gives $E_F \sim \hbar^2(3\pi^2 n)^{2/3}(2m_{\text{eff}})^{-1} \sim 1$ eV for $m_{\text{eff}} \sim m_e$. For the numerical estimate, we use the chemical potential $\mu = 0.3$ eV. Combined with $E_z(w) \sim 16.7$ μeV $(10\text{ nm}/w)^2$, this gives $\delta j/j_0 \sim 8.35 \times 10^{-6}$ $(10\text{ nm}/w)^2$. As representative experimental scales, we consider a film thickness $t = 20$ nm *(62)* and a current density $J = 10^6$ A/cm^2 *(61)*. The background sheet current is $j_0 = Jt \sim 2.0 \times 10^2$ A/m. We use a pickup-loop radius $r_{\text{PL}} = 8$ μm, so that $A_{\text{eff}} \sim \pi(8\ \mu\text{m})^2$, and a sensor height $h = 0.2$ μm *(67)*. With these parameters, the estimated flux is $\Phi \sim 1.63$ $\mu\Phi_0$ $(10\text{ nm}/w)$. The resulting fluxes for different domain wall widths, w, are shown in Table S1. For a sharp wall with $w = 4$ nm, the estimated signal is approximately 4.1 $\mu\Phi_0$, which is comparable or larger than the current-generated magnetic fluxes that have been measured with scanning SQUIDs in other material platforms *(51, 52)*.

S6. INTRINSIC SPIN-ORBIT COUPLING

In this section, we provide more details on the derivation of the effective low-energy Hamiltonian of the altermagnetic texture in the presence of a finite intrinsic spin-orbit coupling.

S6.1. Rotating frame Hamiltonian

We will first define the Hamiltonian of the altermagnetic texture with an intrinsic Rashba spin-orbit coupling and derive its form in the rotating frame.

We start from the Hamiltonian,

$$\hat{\mathcal{H}} = \hat{\varepsilon}_{0,\hat{\boldsymbol{p}}}\tau_0 + \hat{t}_{x,\hat{\boldsymbol{p}}}\tau_x + \hat{t}_{z,\hat{\boldsymbol{p}}}\tau_z + J\tau_z \boldsymbol{n}(\boldsymbol{r})\cdot\boldsymbol{s} + \hat{\mathcal{H}}_{\text{SOC}} \quad \text{with} \quad \hat{\mathcal{H}}_{\text{SOC}} = \alpha_R(\hat{p}_y\tau_x s_x - \hat{p}_x\tau_x s_y), \tag{S96}$$

where α_R is the Rashba spin-orbit coupling strength. It will be useful to write the spin-orbit coupling in the following equivalent form,

$$\hat{\mathcal{H}}_{\text{SOC}} = \frac{\alpha_R}{2}[\{\hat{p}_y, \tau_x s_x\} - \{\hat{p}_x, \tau_x s_y\}]. \tag{S97}$$

We now move to the rotating frame in which the exchange field is aligned with the spin quantization axis. Under the previously defined unitary rotation, $U(\boldsymbol{r})$, the Hamiltonian takes on the form,

$$\hat{\mathcal{H}}_{\text{rot}} = \hat{\varepsilon}_{0,\hat{\boldsymbol{\pi}}}\tau_0 + \hat{t}_{x,\hat{\boldsymbol{\pi}}}\tau_x + \hat{t}_{z,\hat{\boldsymbol{\pi}}}\tau_z + J\tau_z s_z + \hat{\mathcal{H}}_{\text{SOC}}^{\text{rot}} \tag{S98}$$

with the spin-orbit coupling in the rotating frame,

$$\hat{\mathcal{H}}_{\text{SOC}}^{\text{rot}} = \frac{\alpha_R}{2}[\{\hat{\pi}_y, \tau_x\widetilde{s}_x(\boldsymbol{r})\} - \{\hat{\pi}_x, \tau_x\widetilde{s}_y(\boldsymbol{r})\}] \quad \text{where} \quad \widetilde{s}_{x,y,z}(\boldsymbol{r}) = U^\dagger(\boldsymbol{r})s_{x,y,z}U(\boldsymbol{r}). \tag{S99}$$

In addition to making the exchange term spatially uniform, the transformation again has the effect that the momenta are shifted a spin-dependent gauge field, $\hat{\pi}_i$. Moreover, it is again helpful to write these shifted momenta in the form $\hat{\pi}_i = \hat{\Pi}_i - A_i^\perp$. The spin-orbit term can then be written as,

$$\hat{\mathcal{H}}_{\text{SOC}}^{\text{rot}} = \frac{\alpha_R}{2}\tau_x[\{\hat{\Pi}_y, \widetilde{s}_x\} - \{\hat{\Pi}_x, \widetilde{s}_y\}] - \frac{\alpha_R}{2}\tau_x[\{A_y^\perp, \widetilde{s}_x\} - \{A_x^\perp, \widetilde{s}_y\}]. \tag{S100}$$

S6.2. Effective Hamiltonian

We will now project the spin-orbit coupling onto the low-energy subspace with the projector P_-.

For this purpose, we decompose the rotated spin operators into components parallel and perpendicular to the local exchange field, $\widetilde{s}_a = \widetilde{s}_a^\parallel + \widetilde{s}_a^\perp$ with $a = x, y, z$ and $\widetilde{s}_a^\parallel \propto s_z$ and $\widetilde{s}_a^\perp \propto s_x, s_y$.

We now make two helpful observations. First, we note that,

$$\{A_i^\perp, \widetilde{s}_a^\parallel\} = 0, \tag{S101}$$

since $\{s_x, s_z\} = \{s_y, s_z\} = 0$. Second, we note that,

$$P_-\tau_x\{A_i^\perp, \widetilde{s}_a\}P_- = 0. \tag{S102}$$

From the observations, we deduce that only the longitudinally shifted momentum, $\hat{\Pi}_i$, contributes to the projected spin-orbit term,

$$P_-\hat{\mathcal{H}}_{\text{SOC}}^{\text{rot}}P_- = \frac{\alpha_R}{2}[\{\hat{\Pi}_y, \widetilde{\sigma}_x\} - \{\hat{\Pi}_x, \widetilde{\sigma}_y\}]. \tag{S103}$$

Here, we have defined the matrices,

$$\widetilde{\sigma}_a = P_-\,\tau_x\,\widetilde{s}_a^\perp\,P_-, \quad \text{with} \quad a = x, y. \tag{S104}$$

To compute the matrices, we expand the transverse part of the rotated spin operator,

$$\widetilde{s}_a^\perp = \sum_{b=x,y} R_{ab}s_b \quad \text{with} \quad R_{ab} = \frac{1}{2}\,\text{tr}[U^\dagger(\boldsymbol{r})\,s_a\,U(\boldsymbol{r})\,s_b]. \tag{S105}$$

We then find the following expression,

$$\begin{aligned}\widetilde{\sigma}_a &= \sum_{b=x,y} R_{ab}\,P_-\,\tau_x\,s_b\,P_- \\ &= \frac{1}{2}\sum_{b=x,y}\eta_{bb}\,\sigma_b\,\text{tr}[U^\dagger(\boldsymbol{r})s_aU(\boldsymbol{r})s_b].\end{aligned} \tag{S106}$$

Here, we have used that $P_-\tau_x s_x P_- = \sigma_x$ and $P_-\tau_x s_y P_- = -\sigma_y$ and defined, as before, $\eta = \text{diag}(1, -1)$.

S6.3. Radial domain wall

We now focus again on the example of the radial domain wall. We take $\theta(\boldsymbol{r}) = \pi/2$, so that the Néel vector is $\boldsymbol{n}(r) = (\cos\phi(r), \sin\phi(r), 0)$.

We begin by noting that for the radial domain wall texture the transverse components of the rotated spin operators take on the form,

$$\begin{aligned} \widetilde{s}_x^\perp &= \sin\phi\,(\sin\phi\, s_x - \cos\phi\, s_y), \\ \widetilde{s}_y^\perp &= \cos\phi\,(-\sin\phi\, s_x + \cos\phi\, s_y), \end{aligned} \tag{S107}$$

and, similarly,

$$\begin{aligned} \widetilde{\sigma}_x &= \sin\phi\,(\sin\phi\,\sigma_x + \cos\phi\,\sigma_y), \\ \widetilde{\sigma}_y &= -\cos\phi\,(\sin\phi\,\sigma_x + \cos\phi\,\sigma_y). \end{aligned} \tag{S108}$$

We insert these expressions into the projected spin-orbit coupling, which gives the following intermediate result,

$$P_- \hat{\mathcal{H}}_{\mathrm{SOC}}^{\mathrm{rot}} P_- = \frac{\alpha_R}{2}[\{\hat{\Pi}_y, \sin\phi\,(\sin\phi\,\sigma_x + \cos\phi\,\sigma_y)\} + \{\hat{\Pi}_x, \cos\phi\,(\sin\phi\,\sigma_x + \cos\phi\,\sigma_y)\}]. \tag{S109}$$

We can now, effectively, remove the longitudinally shifted momentum from this expression. For this purpose, we note that the longitudinally shifted momentum is,

$$\hat{\Pi}_i = \hat{p}_i - \frac{\hbar}{2}(\partial_i\phi)\,\sigma_z. \tag{S110}$$

In particular, we see that this shift can be removed by the unitary transformation,

$$G^\dagger(\boldsymbol{r})\,\hat{\Pi}_i\,G(\boldsymbol{r}) = \hat{p}_i. \quad \text{with} \quad G(\boldsymbol{r}) = \exp\left[\frac{i}{2}\phi(r)\sigma_z\right]. \tag{S111}$$

For consistency, this transformation must also be applied to the projected spin matrices entering the spin-orbit term. We note that,

$$\begin{aligned} G^\dagger(\boldsymbol{r})\,\widetilde{\sigma}_x\,G(\boldsymbol{r}) &= \sin\phi(r)\,\sigma_y \\ G^\dagger(\boldsymbol{r})\,\widetilde{\sigma}_y\,G(\boldsymbol{r}) &= -\cos\phi(r)\,\sigma_y, \end{aligned} \tag{S112}$$

so that,

$$G^\dagger(P_- \hat{\mathcal{H}}_{\mathrm{SOC}}^{\mathrm{rot}} P_-)G = \frac{\alpha_R}{2}\,\sigma_y[\{\hat{p}_y, \sin\phi\} + \{\hat{p}_x, \cos\phi\}]. \tag{S113}$$

We now combine this simplified projected spin-orbit coupling with the previously obtained projected altermagnetic texture Hamiltonian,

$$\hat{\mathcal{H}}_{\mathrm{rot}}^{(0)} = \hat{\varepsilon}_{0,\hat{\boldsymbol{p}}}\,\sigma_0 + \hat{t}_{z,\hat{\boldsymbol{p}}}\,\sigma_z + \varrho_0\,V_0(\boldsymbol{r})\,\sigma_0 + \varrho_z\,V_z(\boldsymbol{r})\,\sigma_z + \frac{\alpha_R}{2}\,\sigma_y[\{\hat{p}_y, \sin\phi\} + \{\hat{p}_x, \cos\phi\}], \tag{S114}$$

where,

$$\hat{\varepsilon}_{0,\hat{\boldsymbol{p}}} = \varrho_0\,(\hat{p}_x^2 + \hat{p}_y^2), \quad \hat{t}_{z,\hat{\boldsymbol{p}}} = \varrho_z\,(\hat{p}_x^2 - \hat{p}_y^2), \quad V_0(r) = \frac{\hbar^2}{4}\phi'(r)^2, \quad V_z(\boldsymbol{r}) = \frac{\hbar^2}{4}\phi'(r)^2\cos(2\varphi). \tag{S115}$$

In the semiclassical limit, this effective Hamiltonian reduces to,

$$\mathcal{H}_{\mathrm{rot}}^{(0)} = [\varepsilon_{0,\boldsymbol{p}} + \varrho_0\,V_0(r)]\sigma_0 + [t_{z,\boldsymbol{p}} + \varrho_z\,V_z(\boldsymbol{r})]\sigma_z + \alpha_R[p_x\cos\phi(r) + p_y\sin\phi(r)]\sigma_y. \tag{S116}$$

The corresponding local spectrum is then given by,

$$E_\pm(\boldsymbol{r}, \boldsymbol{p}) = \varepsilon_{0,\boldsymbol{p}} + \varrho_0\,V_0(r) \pm \sqrt{[t_{z,\boldsymbol{p}} + \varrho_z\,V_z(\boldsymbol{r})]^2 + \alpha_R^2[p_x\cos\phi(r) + p_y\sin\phi(r)]^2}. \tag{S117}$$

S6.4. Effects on lensing

We next provide details on the effects of intrinsic spin-orbit coupling on our proposed lensing protocols.

The intrinsic Rashba spin-orbit coupling will, in general, mix the two $\tau = \sigma_z$ sectors. However, the intrinsic Rashba spin-orbit coupling is purely off-diagonal in $\mathcal{H}^{(0)}_{\rm rot}$. As a result, it does not couple the two $\tau = \sigma_z$ sectors to first order in α_R,

$$\langle\tau|\sigma_y|\tau\rangle = 0. \tag{S118}$$

As a result, the first spin-orbit induced correction to the local spectrum is second order in α_R. To illustrate this point, we write our effective Hamiltonian in the form,

$$\mathcal{H}^{(0)}_{\rm rot} = h_0(\boldsymbol{r},\boldsymbol{p})\sigma_0 + D(\boldsymbol{r},\boldsymbol{p})\sigma_z + \alpha_R Q(\boldsymbol{r},\boldsymbol{p})\sigma_y, \tag{S119}$$

where we have defined,

$$h_0(\boldsymbol{r},\boldsymbol{p}) = \varepsilon_{0,\boldsymbol{p}} + \varrho_0 V_0(r), \quad D(\boldsymbol{r},\boldsymbol{p}) = t_{z,\boldsymbol{p}} + \varrho_z V_z(\boldsymbol{r}), \quad Q(\boldsymbol{r},\boldsymbol{p}) = p_x\cos\phi(r) + p_y\sin\phi(r). \tag{S120}$$

The local spectrum up to second order in α_R of $\mathcal{H}^{(0)}_{\rm rot}$ is thus given by,

$$E_\tau(\boldsymbol{r},\boldsymbol{p}) \simeq h_0(\boldsymbol{r},\boldsymbol{p}) + \tau D(\boldsymbol{r},\boldsymbol{p}) + \tau\frac{\alpha_R^2 Q(\boldsymbol{r},\boldsymbol{p})^2}{2D(\boldsymbol{r},\boldsymbol{p})} + \ldots \tag{S121}$$

for $\tau = \pm$. In particular, we note from this result that the effect of intrinsic spin-orbit coupling is negligible provided that along any ray of our lensing protocol the condition,

$$\left|\frac{\alpha_R Q(\boldsymbol{r},\boldsymbol{p})}{D(\boldsymbol{r},\boldsymbol{p})}\right| \ll 1 \tag{S122}$$

is satisfied. This condition can be satisfied when $D(\boldsymbol{r},\boldsymbol{p}) \neq 0$ as long as the spin-orbit coupling is sufficiently weak. It is important to note though that even if the spin-orbit coupling is weak, the expansion becomes uncontrolled close to the phase-space manifold defined by,

$$D(\boldsymbol{r},\boldsymbol{p}) = 0. \tag{S123}$$

It is thus important that phase space points that are sufficiently close to this manifold are avoided.

S7. SIMULATION DETAILS

In this section, we provide technical details on our simulations for the figures of the main text.

S7.1. Simulation details for Fig. 2

We first summarize our approach for obtaining the results shown in Fig. 2 of the main text.

In all panels, we consider a static, coplanar circular Néel domain wall with,

$$\theta(\boldsymbol{r}) = \frac{\pi}{2}, \quad \phi(\boldsymbol{r}) = \frac{\pi}{2}\tanh\left(\frac{r-R}{w}\right), \quad \text{and} \quad \phi'(r) = \frac{\pi}{2w}\,\text{sech}^2\left(\frac{r-R}{w}\right), \tag{S124}$$

where we are using polar coordinates, $\boldsymbol{r} = (r,\varphi)$. Since the texture is coplanar and time-independent, the emergent orbital fields vanish, $\mathcal{B}_\tau = \mathcal{E}_{i,\tau} = 0$. We then evaluate the metric tensor of the texture,

$$g_{ij}(\boldsymbol{r}) = \frac{1}{4}\,\partial_i\boldsymbol{n}(\boldsymbol{r})\cdot\partial_j\boldsymbol{n}(\boldsymbol{r}) = \frac{\phi'(r)^2}{4}\,\hat{r}_i\hat{r}_j, \qquad \hat{\boldsymbol{r}} = (\cos\varphi,\sin\varphi). \tag{S125}$$

with $K_\tau^{ij} = \varrho_0 \delta^{ij} + \tau \varrho_z \eta^{ij}$.

For the d-wave altermagnetic texture in *panel (a)*, we plot,

$$V_z(\boldsymbol{r}) = \hbar^2 \eta^{ij} g_{ij}(\boldsymbol{r}) = \hbar^2 [g_{xx}(\boldsymbol{r}) - g_{yy}(\boldsymbol{r})] = (\hbar^2/4)\phi'(r)^2 \cos(2\varphi). \tag{S126}$$

For the g-wave altermagnetic texture in *panel (b)*, we plot,

$$V_z^{(g)}(\boldsymbol{r}) = \hbar^4 T^{ijkl} g_{ij}(\boldsymbol{r}) g_{kl}(\boldsymbol{r}) = \hbar^4 g_{xy}(g_{xx} - g_{yy}) = (\hbar^4/64)\phi'(r)^4 \sin(4\varphi). \tag{S127}$$

The parameters used in the simulations are given in Table S2. We set $\hbar = 1$.

R	w
4.0	0.55

TABLE S2. **Simulation parameters for Fig. 2.**

S7.2. Simulation details for Fig. 3

We next summarize our approach for obtaining the results shown in Fig. 3 of the main text.

In all panels, we consider again a static, coplanar circular Néel domain wall with the same parameterization as in Fig. 3. The effective metric is given by,

$$g^{ij}_{\mathrm{eff},\tau}(\boldsymbol{r}) = K_\tau^{ij} - \frac{2\hbar^2}{J}\left[K_\tau\, g(\boldsymbol{r}) K_\tau\right]^{ij}, \tag{S128}$$

with $K_\tau^{ij} = \varrho_0 \delta^{ij} + \tau \varrho_z \eta^{ij}$

For *panels (a) and (b)*, we compute the eigenvalues $\lambda_{\max}(\boldsymbol{r}) \geq \lambda_{\min}(\boldsymbol{r})$ of $g^{ij}_{\mathrm{eff},\tau}(\boldsymbol{r})$ and plot the anisotropy,

$$\Delta\lambda(\boldsymbol{r}) = \lambda_{\max}(\boldsymbol{r}) - \lambda_{\min}(\boldsymbol{r}), \tag{S129}$$

which we normalize by its maximum value across both τ sectors.

For *panels (c) and (d)*, we evaluate the eigenvector associated with $\lambda_{\max}$ along the domain-wall circle $r = R$. We then sample angles φ on the circle and draw short line segments in the direction of that eigenvector to show the local principal axis orientation. The parameters used in our simulations of Fig. 3 are shown in Table S3, where we have again set $\hbar = 1$.

ϱ_0	ϱ_z	J	R	w
1.0	0.30	10.0	4.0	0.55

TABLE S3. **Simulation parameters for Fig. 3.**

S7.3. Simulation details for Fig. 4

We next summarize our approach for obtaining the results shown in Fig. 4 of the main text.

In all panels, we consider again a static, coplanar circular Néel domain wall with the same parameterization as in Fig. 2 and Fig. 3. The semiclassical dispersion for each $\tau = \pm$ sector is,

$$\varepsilon_\tau(\boldsymbol{p}, \boldsymbol{r}) = \boldsymbol{p}^T\, g^{\mathrm{eff}}_\tau(\boldsymbol{r}) \boldsymbol{p} + V_\tau(\boldsymbol{r}) \quad \text{with} \quad V_\tau(\boldsymbol{r}) = \varrho_0 V_0(\boldsymbol{r}) + \tau \varrho_z V_z(\boldsymbol{r}). \tag{S130}$$

We obtain the ray trajectories from Hamilton's equations,

$$\dot{r}_i = \frac{\partial \varepsilon_\tau}{\partial p_i}, \qquad \dot{p}_i = -\frac{\partial \varepsilon_\tau}{\partial r_i}. \tag{S131}$$

For the simulations, we again set $\hbar = 1$ and use the initial conditions $\boldsymbol{r}_{\text{in}} = (-x_0, b)$ and $\boldsymbol{p}_{\text{in}} = (p_0, 0)$, with several impact parameters $b \in [-2.2, 2.2]$. While we fix the incoming momentum p_0 to be the same for both $\tau = \pm 1$, we remark that the group velocity along the x-direction, $v_\tau = \frac{\partial \varepsilon_\tau}{\partial p_x} = 2(\varrho_0 + \tau \varrho_z) p_0$, will still be τ-dependent. With our simulation parameters, $\varrho_0 = 1$, $\varrho_z = 0.35$, we obtain $v_+/v_- \simeq 2.08$. So the $\tau = +$ rays are more than twice as fast as the $\tau = -$ rays. Because the group velocity is larger for $\tau = +$, the corresponding potential-induced deflection is suppressed.

The parameters used in Fig. 4 are summarized in Table S4.

	ϱ_0	ϱ_z	w	J	Π_0	R
Fig. 4(a,c)	1.0	0.35	0.40	8.7	2.6	4.0
Fig. 4(b,d)	1.0	0.35	0.60	30.0	2.0	4.0

TABLE S4. **Simulation parameters for Fig. 4.**

S7.4. Simulation details for Figure 5 of the main text

We lastly summarize our approach for obtaining the results shown in Fig. 5 of the main text.

In *panel (a)*, we show a circular Néel domain wall with the same parameterization as in Figs. 2-4.

In *panel (b)*, we obtain the constant-energy contour from the local dispersion, $E_\pm(\boldsymbol{p}; \boldsymbol{r}) = \mu$, with,

$$E_\pm(\boldsymbol{p}; \boldsymbol{r}) = \varepsilon_{0,\boldsymbol{p}} + \varrho_0 V_0(\boldsymbol{r}) \pm \sqrt{(t_{z,\boldsymbol{p}} + \varrho_z V_z(\boldsymbol{r}))^2 + \mathrm{g}_{\boldsymbol{p}}^2(\boldsymbol{r})}, \tag{S132}$$

evaluated at the marked point, $(r, \varphi) = (R, \varphi_0)$. Here, $t_{z,\boldsymbol{p}} = \varrho_z(p_x^2 - p_y^2)$ and the spin-orbit field is given by, $\mathrm{g}_{\boldsymbol{p}}(\boldsymbol{r}) = g_0(\sin\theta(\boldsymbol{r})\boldsymbol{p} \cdot \nabla\phi(\boldsymbol{r}), \boldsymbol{p} \cdot \nabla\theta(\boldsymbol{r}))^T$

In *panel (c)*, we plot a skyrmion texture defined by

$$\theta(r) = 2 \arctan\left(\frac{a^2}{r^2}\right) \quad , \quad \phi(\boldsymbol{r}) = \varphi \tag{S133}$$

In *panel (d)*, the constant-energy contours are computed as in panel (b), evaluated at the marked point $(r, \varphi) = (r_0, \varphi_0)$.

The parameters used in our simulations are given in Table S5.

	ϱ_0	ϱ_z	g_0	μ	(r_0, φ_0)	R	w	a
Fig. 5(a,b)	1.0	0.3	-0.4	1.0	$(R, \pi/4)$	3.6	0.7	–
Fig. 5(c,d)	1.0	0.3	-0.4	1.0	$(1.0, \pi/4)$	–	–	2.0

TABLE S5. **Simulation parameters for Fig. 5.**

REFERENCES

1. S. S. Parkin, M. Hayashi, L. Thomas, Magnetic domain-wall racetrack memory. *Science* **320**, 190–194 (2008).

2. A. Fert, V. Cros, J. Sampaio, Skyrmions on the track. *Nat. Nanotechnol.* **8**, 152–156 (2013).

3. N. Morales-Durán, N. Wei, J. Shi, A. H. MacDonald, Magic angles and fractional Chern insulators in twisted homobilayer transition metal dichalcogenides. *Phys. Rev. Lett.* **132**, 096602 (2024).

4. J. Shi, N. Morales-Durán, E. Khalaf, A. H. MacDonald, Adiabatic approximation and Aharonov-Casher bands in twisted homobilayer transition metal dichalcogenides. *Phys. Rev. B* **110**, 035130 (2024).

5. K. c. v. Kolář, K. Yang, F. von Oppen, C. Mora, Hofstadter spectrum of Chern bands in twisted transition metal dichalcogenides. *Phys. Rev. B* **110**, 115114 (2024).

6. B. Li, F. Wu, Variational mapping of Chern bands to Landau levels: Application to fractional Chern insulators in twistedMoTe2. *Phys. Rev. B* **111**, 125122 (2025).

7. D. Guerci, A. Abouelkomsan, L. Fu, Topological superconductivity with emergent vortex lattice in twisted semiconductors. arXiv:2602.15106 [cond-mat.supr-con] (2026).

8. G. E. Volovik, Linear momentum in ferromagnets. *J. Phys. C: Solid State Phys.* **20**, L83–L87 (1987).

9. P. Bruno, V. K. Dugaev, M. Taillefumier, Topological Hall effect and Berry phase in magnetic nanostructures. *Phys. Rev. Lett.* **93**, 096806 (2004).

10. S. A. Yang, G. S. D. Beach, C. Knutson, D. Xiao, Q. Niu, M. Tsoi, J. L. Erskine, Universal electromotive force induced by domain wall motion. *Phys. Rev. Lett.* **102**, 067201 (2009).

11. P. N. Hai, S. Ohya, M. Tanaka, S. E. Barnes, S. Maekawa, Electromotive force and huge magnetoresistance in magnetic tunnel junctions. *Nature* **458**, 489–492 (2009).

12. S. E. Barnes, S. Maekawa, Generalization of Faraday's law to include nonconservative spin forces. *Phys. Rev. Lett.* **98**, 246601 (2007).

13. T. Schulz, R. Ritz, A. Bauer, M. Halder, M. Wagner, C. Franz, C. Pfleiderer, K. Everschor, M. Garst, A. Rosch, Emergent electrodynamics of skyrmions in a chiral magnet. *Nat. Phys.* **8**, 301–304 (2012).

14. Y. Onishi, N. Paul, L. Fu, Emergent curved space and gravitational lensing in quantum materials. *Phys. Rev. B* **113**, 024401 (2026).

15. R. Cheng, Q. Niu, Electron dynamics in slowly varying antiferromagnetic texture. *Phys. Rev. B* **86**, 245118 (2012).

16. O. Gomonay, Berry-phase effects and electronic dynamics in a noncollinear antiferromagnetic texture. *Phys. Rev. B* **91**, 144421 (2015).

17. A. Okabayashi, T. Morinari, Theory of spin motive force in one-dimensional antiferromagnetic domain wall. *J. Physical Soc. Japan* **84**, 033706 (2015).

18. Y. Yamane, J. Ieda, J. Sinova, Electric voltage generation by antiferromagnetic dynamics. *Phys. Rev. B* **93**, 180408 (2016).

19. A. Manchon, J. Železný, I. M. Miron, T. Jungwirth, A. Sinova, K. G. Thiaville, P. Gambardella, Current-induced spin-orbit torques in ferromagnetic and antiferromagnetic systems. *Rev. Mod. Phys.* **91**, 035004 (2019).

20. S. Zhang, Z. Li, Roles of nonequilibrium conduction electrons on the magnetization dynamics of ferromagnets. *Phys. Rev. Lett.* **93**, 127204 (2004).

21. Y. Xu, S. Wang, K. Xia, Spin-Transfer Torques in Antiferromagnetic Metals from First Principles. *Phys. Rev. Lett.* **100**, 226602 (2008).

22. A. C. Swaving, R. A. Duine, Current-induced torques in continuous antiferromagnetic textures. *Phys. Rev. B* **83**, 054428 (2011).

23. K. M. D. Hals, Y. Tserkovnyak, A. Brataas, Phenomenology of current-induced dynamics in antiferromagnets. *Phys. Rev. Lett.* **106**, 107206 (2011).

24. Y. Yamane, J. Ieda, J. Sinova, Spin-transfer torques in antiferromagnetic textures: Efficiency and quantification method. *Phys. Rev. B* **94**, 054409 (2016).

25. I. Žutić, J. Fabian, S. Das Sarma, Spintronics: Fundamentals and applications. *Rev. Mod. Phys.* **76**, 323–410 (2004).

26. V. Baltz, A. Manchon, M. Tsoi, T. Moriyama, T. Ono, Y. Tserkovnyak, Antiferromagnetic spintronics. *Rev. Mod. Phys.* **90**, 015005 (2018).

27. T. Jungwirth, J. Sinova, P. Wadley, D. Kriegner, H. Reichlova, F. Krizek, H. Ohno, L. Smejkal, Altermagnetic spintronics. arXiv:2508.09748 [cond-mat.mtrl-sci] (2025).

28. L. Šmejkal, J. Sinova, T. Jungwirth, Emerging research landscape of altermagnetism. *Phys. Rev. X* **12**, 040501 (2022).

29. S. S. Fender, O. Gonzalez, D. K. Bediako, Altermagnetism: A chemical perspective. *J. Am. Chem. Soc.* **147**, 2257–2274 (2025).

30. R. Tamang, S. Gurung, D. P. Rai, S. Brahimi, S. Lounis, Altermagnetism and altermagnets: A brief review. *Magnetism* **5**, 17 (2025).

31. I. Mazin, The PRX Editors, Editorial: Altermagnetism—A new punch line of fundamental magnetism. *Phys. Rev. X* **12**, 040002 (2022).

32. T. Jungwirth, R. M. Fernandes, E. Fradkin, A. H. MacDonald, J. Sinova, L. Šmejkal, Altermagnetism: An unconventional spin-ordered phase of matter. *Newton* **1**, 100162 (2025).

33. R. Yamamoto, L. A. Turnbull, M. Schmidt, J. C. Cor-saletti Filho, H. J. Binger, M. Di Pietro Martínez, M. Weigand, S. Finizio, Y. Prots, G. M. Ferguson, U. Vool, S. Wintz, C. Donnelly, Altermagnetic nanotextures revealed in bulk MnTe. *Phys. Rev. Appl.* **24**, 034037 (2025).

34. O. J. Amin, A. D. Din, E. Golias, Y. Niu, A. Zakharov, S. C. Fromage, C. J. B. Fields, S. L. Heywood, R. B. Cousins, F. Maccherozzi, J. Krempaský, J. H. Dil, D. Kriegner, B. Kiraly, R. P. Campion, A. W. Rushforth, K. W. Edmonds, S. S. Dhesi, L. Šmejkal, T. Jungwirth, P. Wadley, Nanoscale imaging and control of altermagnetism in MnTe. *Nature* **636**, 348–353 (2024).

35. O. Gomonay, V. P. Kravchuk, R. Jaeschke-Ubiergo, K. V. Yershov, T. Jungwirth, L. Šmejkal, J. van den Brink, J. Sinova, Structure, control, and dynamics of altermagnetic textures. *npj Spintron.* **2**, 35 (2024).

36. Z. Jin, Z. Zeng, Y. Cao, P. Yan, Skyrmion Hall effect in altermagnets. *Phys. Rev. Lett.* **133**, 196701 (2024).

37. H. Vakili, E. Schwartz, A. A. Kovalev, Spin-transfer torque in altermagnets with magnetic textures. *Phys. Rev. Lett.* **134**, 176401 (2025).

38. R. Zarzuela, R. Jaeschke-Ubiergo, O. Gomonay, L. Šmejkal, J. Sinova, Transport theory and spin-transfer physics □-wave altermagnets. *Phys. Rev. B* **111**, 064422 (2025).

39. J. Gondolf, A. Kreisel, M. Roig, Y. Yu, D. F. Agterberg, B. M. Andersen, Local signatures of altermagnetism. *Phys. Rev. B* **111**, 174436 (2025).

40. A. Maiani, R. S. Souto, Impurity states in altermagnetic superconductors. *Phys. Rev. B* **111**, 224506 (2025).

41. P. Sukhachov, J. Linder, Impurity-induced Friedel oscillations in altermagnets andp-wave magnets. *Phys. Rev. B* **110**, 205114 (2024).

42. H.-R. Hu, X. Wan, W. Chen, Quasiparticle interference in altermagnets. *Phys. Rev. B* **111**, 035132 (2025).

43. W. Chen, X. Zhou, D. Zhang, Y.-Q. Xu, W.-K. Lou, Impurity scattering and Friedel oscillations in altermagnets. *Phys. Rev. B* **110**, 165413 (2024).

44. M. M. Vasiakin, A. S. Mel'nikov, Disorder-enhanced superconductivity in altermagnet-superconductor hybrids. *Phys. Rev. B* **111**, L100502 (2025).

45. G. A. Bobkov, I. V. Bobkova, A. M. Bobkov, Proximity effect in superconductor/antiferromagnet hybrids: Néel triplets and impurity suppression of superconductivity. *Phys. Rev. B* **108**, 054510 (2023).

46. D. Sedov, M. S. Scheurer, Quantum geometry and impurity sensitivity of superconductors without time-reversal symmetry: Application to rhombohedral graphene and altermagnets. arXiv:2510.19943 [cond-mat.supr-con] (2025).

47. H. Radhakrishnan, B. Bell, C. Ortix, J. W. F. Venderbos, Topological piezomagnetic effect in two-dimensional Dirac quadrupole altermagnets. arXiv:2602.05894 [cond-mat.str-el] (2026).

48. J. W. F. Venderbos, Berry phase polarization and orbital magnetization responses of insulators: Formulas for generalized polarizabilities and their application. arXiv:2512.19380 [cond-mat.other] (2025).

49. M. Roig, A. Kreisel, Y. Yu, B. M. Andersen, D. F. Agterberg, Minimal models for altermagnetism. *Phys. Rev. B* **110**, 144412 (2024).

50. M. Roig, Y. Yu, R. C. Ekman, A. Kreisel, B. M. Andersen, D. F. Agterberg, Quasisymmetry-constrained spin ferromagnetism in altermagnets. *Phys. Rev. Lett.* **135**, 016703 (2025).

51. K. C. Nowack, E. M. Spanton, M. Baenninger, M. König, J. R. Kirtley, B. Kalisky, C. Ames, P. Leubner, C. Brüne, H. Buhmann, L. W. Molenkamp, D. Goldhaber-Gordon, K. A. Moler, Imaging currents in HgTe quantum wells in the quantum spin Hall regime. *Nat. Mater.* **12**, 787–791 (2013).

52. E. M. Spanton, K. C. Nowack, L. Du, G. Sullivan, R.-R. Du, K. A. Moler, Images of edge current inInAs/GaSb quantum wells. *Phys. Rev. Lett.* **113**, 026804 (2014).

53. T. L. Curtright, D. B. Fairlie, C. K. Zachos, *A Concise Treatise on Quantum Mechanics in Phase Space* (World Scientific Publishing, Singapore, 2014).

54. C. Wu, S.-C. Zhang, Dynamic generation of spin-orbit coupling. *Phys. Rev. Lett.* **93**, 036403 (2004).

55. C. Wu, K. Sun, E. Fradkin, S.-C. Zhang, Fermi liquid instabilities in the spin channel. *Phys. Rev. B* **75**, 115103 (2007).

56. E. I. Kiselev, M. S. Scheurer, P. Wölfle, J. Schmalian, Limits on dynamically generated spin-orbit coupling: Absence of l=1Pomeranchuk instabilities in metals. *Phys. Rev. B* **95**, 125122 (2017).

57. A. B. Hellenes, T. Jungwirth, R. Jaeschke-Ubiergo, A. Chakraborty, J. Sinova, L. Šmejkal, P-wave magnets. arXiv:2309.01607 [cond-mat.mes-hall] (2023).

58. A. Maiani, K. Flensberg, M. Leijnse, Conductivity anisotropy and linear dichroism in spin-textured altermagnets. arXiv:2602.14950 [cond-mat.mes-hall] (2026).

59. H. Reichlová, R. Lopes Seeger, R. González-Hernández, I. Kounta, R. Schlitz, D. Kriegner, P. Ritzinger, M. Lammel, M. Leiviskä, V. Petříček, P. Doležal, E. Schmoranzerová, J. Badura, A. Thomas, V. Baltz, L. Šmejkal, S. T. B. Goennenwein, J. Sinova, Observation of a spontaneous anomalous Hall response in the Mn_5Si_3 d-wave altermagnet candidate. *Nat. Commun.* **15**, 4961 (2024).

60. M. Leiviskä, J. Rial, A. Bad'ura, R. L. Seeger, I. Kounta, S. Beckert, D. Kriegner, I. Joumard, E. Schmoranzerová, J. Sinova, O. Gomonay, A. Thomas, S. T. B. Goennenwein, H. Reichlová, L. Šmejkal, L. Michez, T. Jungwirth, V. Baltz, Anisotropy of the anomalous Hall effect in thin films of the altermagnet candidateMn_5Si_3. *Phys. Rev. B* **109**, 224430 (2024).

61. J. Rial, M. Leiviskä, G. Skobjin, A. Bad’ura, G. Gaudin, F. Disdier, R. Schlitz, I. Kounta, S. Beckert, D. Kriegner, A. Thomas, E. Schmoranzerová, L. Šmejkal, J. Sinova, T. c. v. Jungwirth, L. Michez, H. Reichlová, S. T. B. Goennenwein, O. Gomonay, V. Baltz, Altermagnetic variants in thin films of Mn_5Si_3. *Phys. Rev. B* **110**, L220411 (2024).

62. A. Badura, W. H. Campos, V. K. Bharadwaj, I. Kounta, L. Michez, M. Petit, J. Rial, M. Leiviskä, V. Baltz, F. Krizek, D. Kriegner, J. Železný, J. Zemen, S. Telkamp, S. Sailler, M.

Lammel, R. Jaeschke-Ubiergo, A. B. Hellenes, R. González-Hernández, J. Sinova, T. Jungwirth, S. T. B. Goennenwein, L. Šmejkal, H. Reichlova, Observation of the anomalous Nernst effect in altermagnetic candidate Mn_5Si_3. *Nat. Commun.* **16**, 7111 (2025).

63. J. Krempaský, L. Šmejkal, S. W. D'Souza, M. Hajlaoui, G. Springholz, K. Uhlířová, F. Alarab, P. C. Constantinou, V. Strocov, D. Usanov, W. R. Pudelko, R. González-Hernández, A. B. Hellenes, Z. Jansa, H. Reichlová, Z. Šobáň, R. D. G. Betancourt, P. Wadley, J. Sinova, D. Kriegner, J. Minár, J. H. Dil, T. Jungwirth, Altermagnetic lifting of Kramers spin degeneracy. *Nature* **626**, 517–522 (2024).

64. T. Osumi, S. Souma, T. Aoyama, K. Yamauchi, Honma, K. Nakayama, T. Takahashi, K. Ohgushi, T. Sato, Observation of a giant band splitting in altermagnetic MnTe. *Phys. Rev. B* **109**, 115102 (2024).

65. F. Krizek, S. Reimers, Z. Kašpar, A. Marmodoro, O. M. Michalička, A. Edström, O. J. Amin, W. Edmonds, R. P. Campion, F. Maccherozzi, S. S. Dhesi, J. Zubáč, D. Kriegner, D. Carbone, J. Železný, K. Výborný, K. Olejník, V. Novák, J. Rusz, J.-C. Idrobo, P. Wadley, T. Jung-wirth, Atomically sharp domain walls in an antiferromagnet. *Sci. Adv.* **8**, eabn3535 (2022).

66. H. Noad, C. A. Watson, H. Inoue, M. Kim, H. K. Sato, C. Bell, H. Y. Hwang, J. R. Kirtley, K. A. Moler, Observation of signatures of subresolution defects in two-dimensional superconductors with a scanning SQUID. *Phys. Rev. B* **98**, 064510 (2018).

67. X. Wang, A. Kundu, B. Xu, S. Hameed, N. Rothem, S. Rabkin, L. Rogić, L. Thompson, A. McLeod, M. Greven, D. Pelc, I. Sochnikov, B. Kalisky, A. Klein, Multiferroicity in plastically deformed $SrTiO_3$. *Nat. Commun.* **15**, 7442 (2024).
